# Multi-ancestry colocalization approaches

**DOI:** 10.1371/journal.pgen.1012221

**Published:** 2026-07-21

**Authors:** Cathy Shen, Josée Dupuis, Qihuang Zhang

**Affiliations:** Department of Epidemiology, Biostatistics and Occupational Health, McGill University, Montreal, Quebec, Canada; Yale University, UNITED STATES OF AMERICA

## Abstract

Genome-wide association studies (GWAS) have identified thousands of variants associated with complex traits, but many are non-causal. Statistical fine-mapping methods aim to pinpoint the most likely causal variants among the many associated ones. While most fine-mapping methods were originally limited to single ancestry analysis, multi-ancestry fine-mapping methods are now available, leveraging differences in linkage disequilibrium (LD) and minor allele frequencies (MAFs) across ancestries to improve fine-mapping resolution. However, the biological relevance of the putative causal variants identified through fine-mapping often remains unclear. Colocalization methods improve interpretability by integrating GWAS data with other functional genomics datasets to assess whether two traits share the same causal variants. Despite the growing availability of multi-ancestry data, there are currently no established methods for multi-ancestry colocalization. In this study, we propose multi-ancestry colocalization approaches through the integration of multi-ancestry fine-mapping methods, SuSiEx and MsCAVIAR, with single ancestry colocalization methods, coloc and eCAVIAR. We introduce coloc_SuSiEx, eCAVIAR_SuSiEx, eMsCAVIAR and coloc_MsCAVIAR. The performance of the proposed approaches is evaluated and compared through simulation studies. In loci with a single causal variant, credible set sizes across the four approaches were comparable, as was the prioritization of the true causal variant. MsCAVIAR-based approaches were more computationally expensive compared to SuSiEx-based approaches, which is an important consideration for the analysis of regions with multiple causal variants. Compared to the coloc-based approaches, the eCAVIAR-based approaches tended to report lower loci level colocalization posterior probabilities. For the analysis of loci with multiple causal variants, coloc_SuSiEx is the preferred approach. We apply the proposed approaches to perform a colocalization analysis of multi-ancestry T2D GWAS data from the DIAMANTE Consortium and European pQTL data from the INTERVAL study. This work addresses the increasing need for multi-ancestry approaches to colocalization analysis as more multi-ancestry data become available.

## Author summary

Through genome-wide association studies (GWAS), thousands of genetic variants associated with complex traits have been identified. Pinpointing the causal variants among the numerous associated ones is a challenge that statistical fine-mapping methods aim to solve. Colocalization is another common downstream analysis and it assesses whether two traits share the same causal variant. As data from diverse ancestries become more widely available, there is a growing need for multi-ancestry methods. However, there are currently no established methods in the literature for multi-ancestry colocalization analysis. To bridge this gap, we introduce four multi-ancestry colocalization approaches: coloc_SuSiEx, eCAVIAR_SuSiEx, eMsCAVIAR and coloc_MsCAVIAR. These approaches are developed through the cross-integration of the multi-ancestry fine-mapping methods, SuSiEx and MsCAVIAR, with single ancestry colocalization methods, coloc and eCAVIAR. The performance of the proposed approaches is investigated through extensive simulation studies, and their applicability is demonstrated through a colocalization analysis of a multi-ancestry T2D GWAS dataset with a European pQTL dataset.

## Introduction

Genome-wide association studies (GWAS) have identified thousands of genetic variants associated with complex traits, however, most of these variants are non-causal. Due to linkage disequilibrium (LD), it is challenging to pinpoint the variants with a direct effect on the outcome. Statistical fine-mapping methods aim to differentiate causal variants from correlated tagging variants. While most fine-mapping methods are restricted to single ancestry analysis, some have been extended to support multi-ancestry analysis, such as SuSiE/SuSiEx [1,2] and CAVIAR/MsCAVIAR [3,4]. Multi-ancestry analysis can improve fine-mapping resolution by leveraging differences in LD structure and minor allele frequencies (MAFs) between ancestries, as well as increased power from larger sample sizes.

The biological interpretation of fine-mapping results is often challenging, especially if the identified putative causal variants are located in non-coding regions of the genome. The integration of GWAS data with other functional genomics datasets, such as expression quantitative trait loci (eQTL) data, can improve results interpretability. Colocalization analysis is one such way to integrate a GWAS and an eQTL dataset. The aim is to assess whether the association signal observed at the same locus for both traits can be explained by a shared causal variant. A colocalized GWAS-GWAS signal may suggest that the shared variant influences both traits through a common pathway, or a colocalized GWAS-eQTL signal may implicate the gene in the disease mechanism.

Current methods for colocalization analysis include coloc, coloc_SuSiE, and eCAVIAR, among others [5–7]. coloc defines a configuration as the pair of binary vectors (one for each trait) indicating the causal status of each variant. Assuming at most one causal variant per locus, each configuration corresponds with one of the following locus level hypotheses shown in Table 1 [5]. Among these locus level hypotheses, the posterior probability of *H*_4_ (*PP*.*H*4) is of particular interest, as it is the locus level colocalization posterior probability (lCLPP). In addition to the lCLPP, coloc also reports the conditional variant level colocalization posterior probability (cvCLPP). This is the probability that the variant is causal, given that *H*_4_ is true.

**Table 1:** Colocalization hypotheses considered by the coloc method.

Hypothesis	Interpretation
*H*_0_	No association with either trait
*H*_1_	Association with trait 1, not trait 2
*H*_2_	Association with trait 2, not trait 1
*H*_3_	Association with both traits, distinct variants
*H*_4_	Association with both traits, shared variant

The single causal variant assumption is convenient, but it is a limitation of the coloc method, motivating the development of coloc_SuSiE and eCAVIAR to relax this assumption. coloc_SuSiE is an extension of the coloc method that allows for multiple causal variants per locus. Fine-mapping by SuSiE [1,8] can identify multiple causal variants for each trait, and each causal variant is returned in a distinct credible set. coloc subsequently analyzes each pair of signals from trait 1 and trait 2 to assess for colocalization.

**Fig 1:**
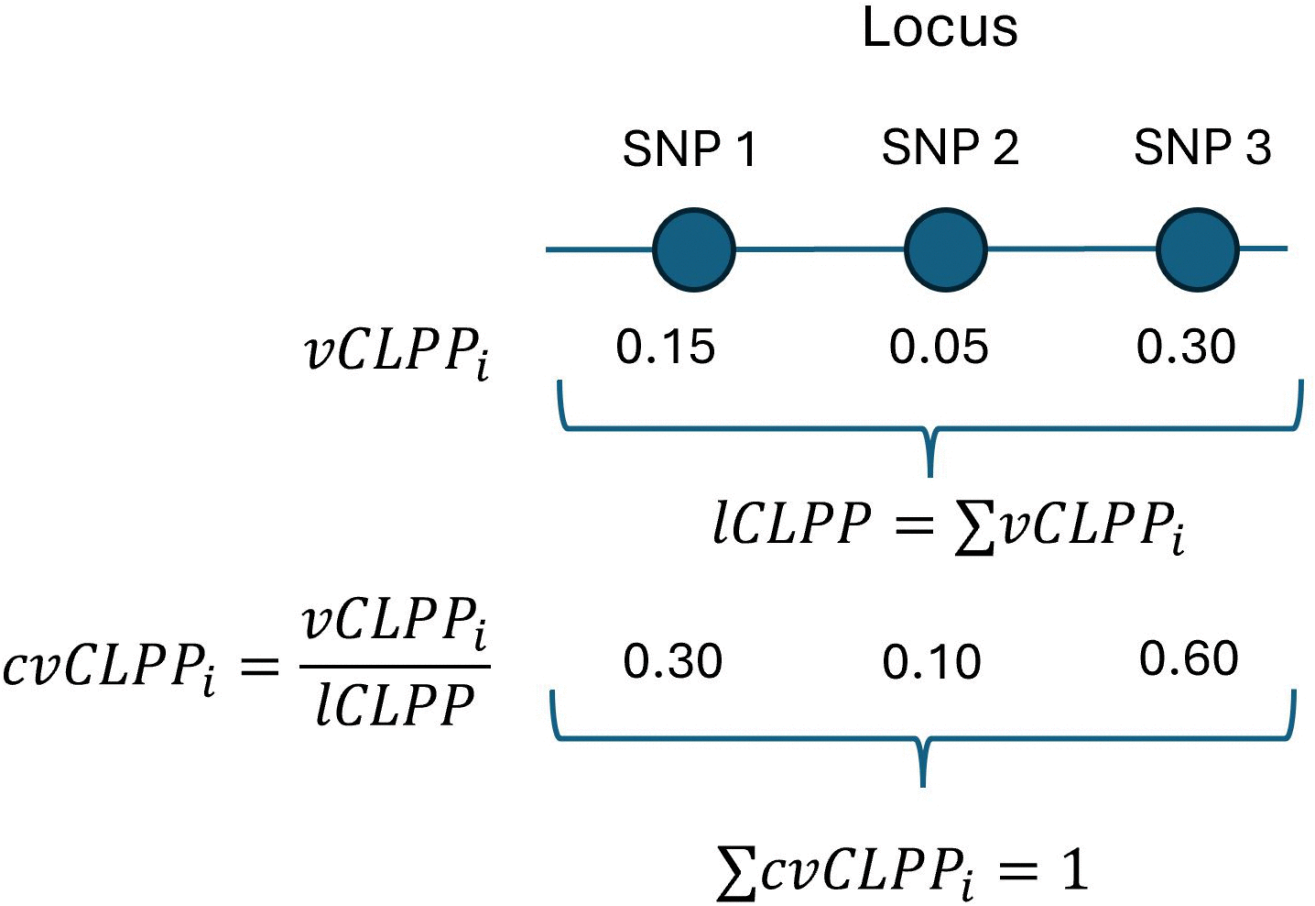
Overview of lCLPP, vCLPP and cvCLPP. Each SNP within a locus is assigned a variant level colocalization posterior probability (vCLPP). Under the assumption of a single colocalized variant, the locus level colocalization posterior probability (lCLPP) is defined as the sum of the vCLPP values across all variants in the locus, representing the overall probability that the locus has a shared causal signal. The conditional variant level colocalization posterior probability (cvCLPP) is calculated for each SNP by normalizing each vCLPP by the lCLPP, representing the probability that a variant is causal given that the locus is colocalized.

eCAVIAR is a colocalization method based on the CAVIAR fine-mapping framework which computes the posterior probability of causality for each variant and for each trait. Under the assumption that the probability of a variant being causal for one trait is independent of it being causal for the other trait analyzed, eCAVIAR computes a variant level colocalization posterior probability (vCLPP). For each variant, this value is computed by multiplying its causal posterior probability for trait 1 by its causal posterior probability for trait 2 [7]. The conditional variant level CLPP (cvCLPP) – which is the probability that a variant is causal given that the locus is colocalized – can be taken as the vCLPP normalized by the locus level colocalization posterior probability (lCLPP). For multi-ancestry colocalization approaches involving coloc or SuSiEx, the lCLPP can simply be taken as the sum of the vCLPPs, as these approaches consider credible sets containing at most one causal variant. For eMsCAVIAR, we define the lCLPP as 1−∏(1−vCLPP) to allow for the possibility of more than one colocalized variant in the locus. The CLPP definitions are summarized by an example illustrated in Fig 1.

A limitation of these colocalization methods is that they are only applicable to single ancestry analyses. These methods were developed under the assumption that the association studies for trait 1 and trait 2 are taken over the same population such that the LD patterns and MAFs are similar. Thus, post-hoc methods for multi-ancestry analysis would ignore the unique LD structures across populations. As with fine-mapping, leveraging differences across ancestries and greater sample sizes could improve colocalization performance. In this study, we integrate multi-ancestry fine-mapping methods, SuSiEx and MsCAVIAR with single ancestry colocalization methods, coloc and eCAVIAR to produce four multi-ancestry colocalization approaches: coloc_SuSiex, eCAVIAR_SuSiEx, eMsCAVIAR, and coloc_MsCAVIAR. The performance of the proposed approaches are evaluated through extensive simulation studies, and we apply each approach to colocalize a multi-ancestry T2D GWAS from the DIAMANTE Consortium [9] with European protein quantitative trait loci (pQTL) data from the INTERVAL study [10].

## Results

### Overview of multi-ancestry colocalization approaches

The proposed multi-ancestry colocalization approaches follow a two step procedure. Multi-ancestry fine-mapping is performed in step 1, using SuSiEx or MsCAVIAR. In this step, ancestry specific summary statistics along with corresponding LD reference panels will be provided as inputs for fine-mapping analysis. Both methods will output fine-mapping credible sets, and the probability of each variant to be included in the credible set. However, the fine-mapping credible set definitions differ. Whereas SuSiEx aims to capture at least one causal variant in each credible set (there can be multiple), MsCAVIAR aims to capture all causal variants in one credible set. These outputs from fine-mapping are subsequently provided to coloc or eCAVIAR for the colocalization analysis in step 2. LD reference information is no longer required at this stage. The colocalization step returns the lCLPP (the colocalization posterior probability of the locus) and the cvCLPP (the variant level colocalization posterior probabilities given that the locus is colocalized). Colocalization credible sets can be constructed from the cvCLPPs. Simulation studies evaluate the performance of coloc_SuSiEx, eMsCAVIAR, eCAVIAR_SuSiEx and coloc_MsCAVIAR, comparing 2 metrics relating to credible sets (size and coverage), and 2 metrics relating to CLPPs (cvCLPPs and lCLPPs).

### One causal variant

#### Multi-ancestry colocalization analysis: an example.

**Fig 2:**
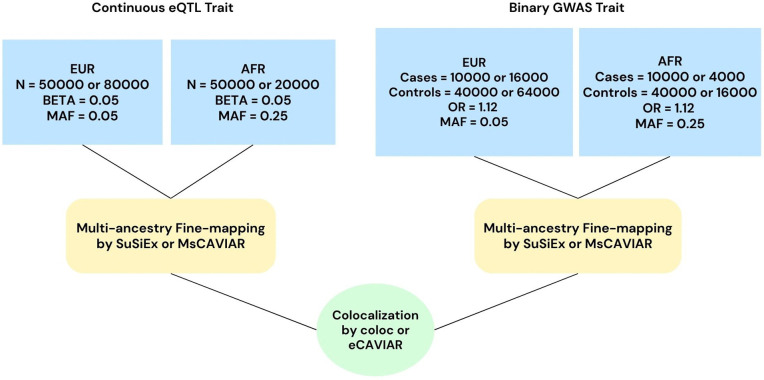
Workflow for simulation study of region chr2:1372831-1622831 (GRCh37). Summary statistics for a continuous (eQTL) trait were simulated for EUR and AFR ancestries using GWASBrewer, such that there is one causal variant rs10189329:1497831:G:A in both ancestries. Similarly, summary statistics for a binary (GWAS) trait were also simulated for EUR and AFR ancestries using simGWAS, with rs10189329:1497831:G:A as the causal variant. MsCAVIAR or SuSiEx performs multi-ancestry fine-mapping of the ancestry stratified summary statistics, those results are provided to coloc or eCAVIAR for subsequent colocalization analysis.

**Fig 3:**
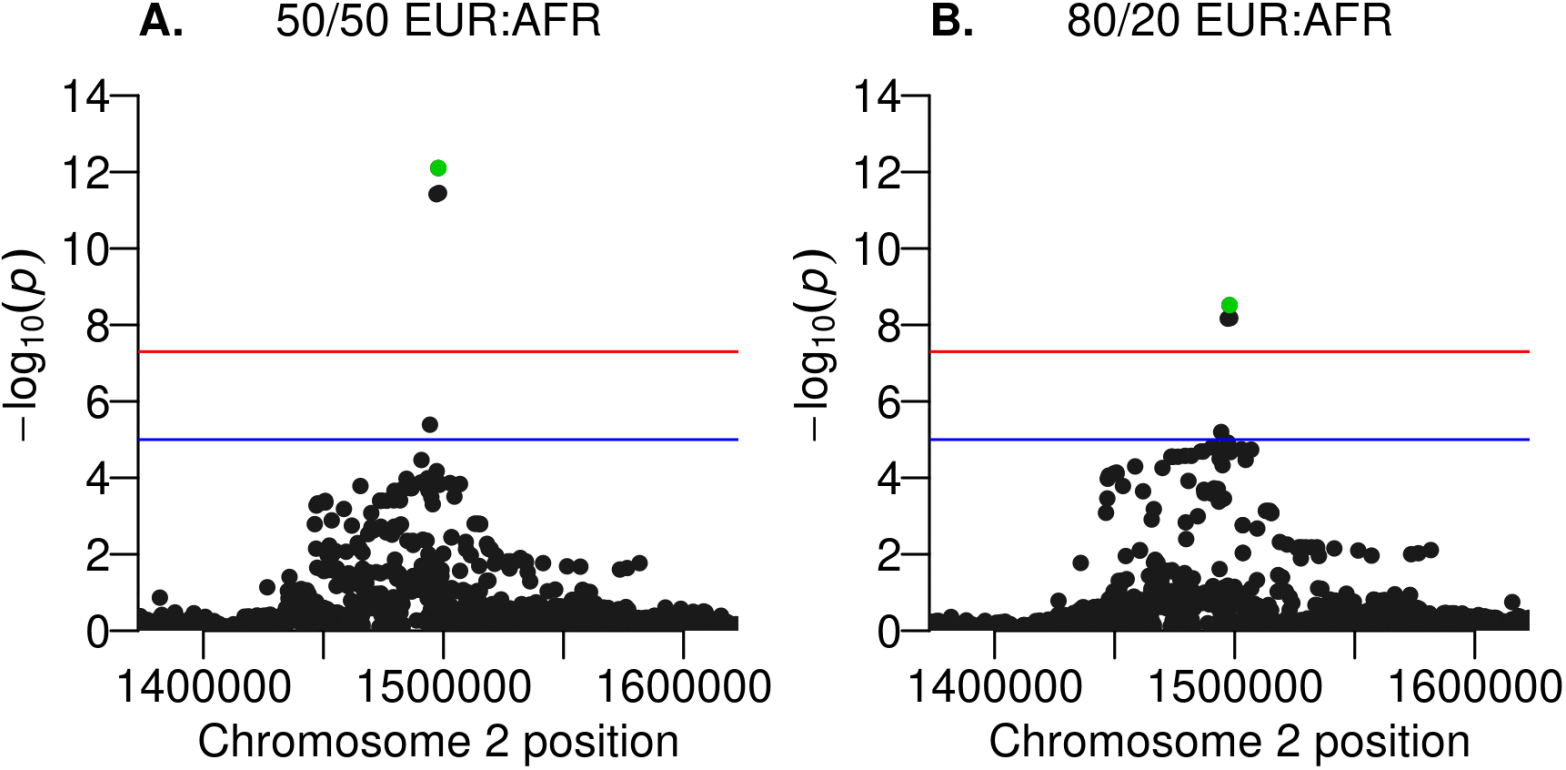
Manhattan plots of $-log_{10}$ p-values of expected z-scores calculated by simGWAS for a GWAS of a binary trait. The region is 1372831-1622831 on chromosome 2 (GRCh37), centered around causal variant rs10189329:1497831:G:A, specified to have an OR of 1.12 in each ancestry. The MAF of the causal variant is 0.05 and 0.25 in the EUR and AFR ancestries, respectively. A 1:4 case-control ratio was specified for both ancestries. SNP p-values from a fixed effects meta analysis over both ancestries are plotted in **A**, with $N_{EUR}=50000,N_{AFR}=50000$ and **B**, with $N_{EUR}=80000,N_{AFR}=20000$, respectively.

In Fig 2, we illustrate an example of a simulation study workflow for a locus under study. Summary statistics for a GWAS of a binary and a continuous trait were simulated for both EUR and AFR ancestry groups. Two different ancestry proportions were considered such that the GWAS included 100,000 participants, either split 50/50 EUR:AFR or 80/20 EUR:AFR. In this example, one variant was selected to be causal. Manhattan plots of the meta-analyzed simulated data for the binary trait are shown in Fig 3. The plot is similar for the quantitative trait, see S1 Fig.

**Fig 4:**
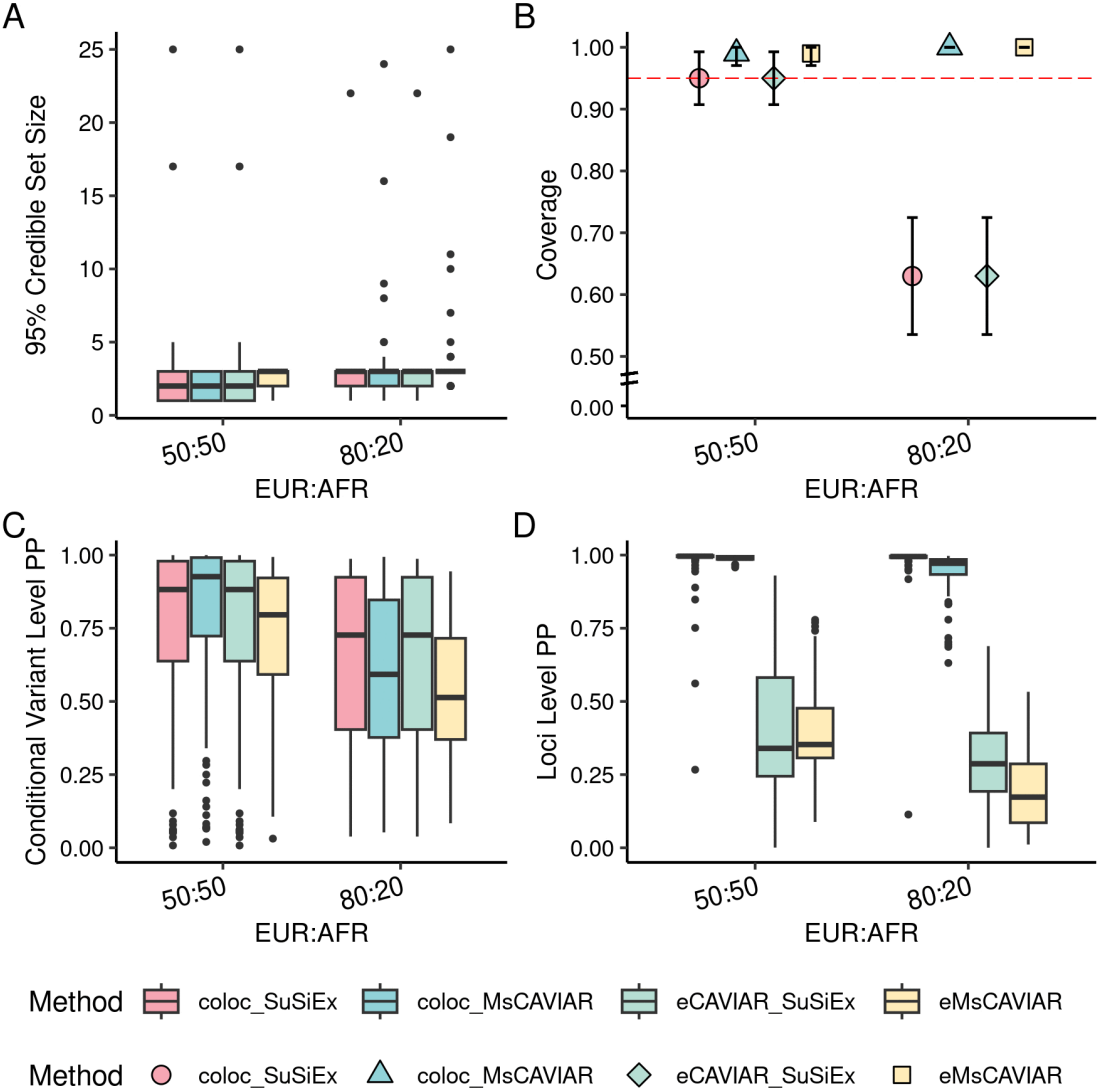
Multi-ancestry colocalization results on simulated region chr2:1372831-1622831 (GRCh37). **A.** 95% colocalization credible sets are constructed by ranking variants by conditional variant level CLPPs and summing until the cumulative CLPP exceeds 0.95. **B.** Coverage is the proportion of iterations the causal variant was included in the 95% colocalization credible set out of the iterations that converged. **C.** The probability that the variant, rs10189329:1497831:G:A is causal given that this locus is colocalized. **D.** The probability of colocalization within the locus.

The ancestry specific summary statistics were then provided to SuSiEx or MsCAVIAR for multi-ancestry fine-mapping. Fine-mapping results were subsequently provided to coloc or eCAVIAR to perform the colocalization analysis. The performance of coloc_SuSiEx, eMsCAVIAR, eCAVIAR_SuSiEx and coloc_MsCAVIAR were evaluated based on the lCLPPs, cvCLPPs of the causal variants, the 95% credible set sizes as well as the inclusion of the causal variants in the credible sets. For this region, credible set sizes, coverage and cvCLPPs of the causal variant are comparable across all approaches. The reported lCLPP differ across approaches. These results are presented in Fig 4.

#### Credible set sizes are comparable across all approaches.

In the simulation study of the region, chr2:1372831–162283 (GRCh37), the median sizes of the 95% colocalization credible sets constructed by the four approaches are comparable within each ancestry proportion (Fig 4A). The median colocalization credible set sizes in this region are larger in the 80/20 EUR:AFR setting compared to the 50/50 EUR:AFR setting (Fig 4A). The MAF of the causal variant, rs10189329:1497831:G:A, was 0.05 and 0.25 in the EUR and AFR ancestries, respectively. The decreased AFR sample size under the 80/20 EUR:AFR setting resulted in a weaker signal from the true causal variant, less certainty on which variant to prioritize, and larger credible set sizes.

We conducted simulation studies on other regions that were specified to have one colocalized variant across both traits, using data from the EUR and AFR ancestries. Median sizes of the 95% colocalization credible sets constructed by the four approaches are comparable within the 50/50 EUR:AFR setting, and within the 80/20 EUR:AFR setting (S2 and S6 Figs). Larger credible set sizes under the uneven ancestry proportion (80/20 EUR:AFR) compared to the even ancestry proportion (50/50 EUR:AFR) are observed in loci where the causal variant MAF in EUR was lower than the MAF in AFR (S5 and S6 Tables).

We conducted simulation studies using data from EUR, EAS and AFR, to assess whether the approaches are robust to analyzing inputs from multiple ancestries. Median credible set sizes are comparable within the 33/33/33 EUR:EAS:AFR setting, and the 75/15/10 EUR:EAS:AFR setting (S10 Fig). Once again, larger credible set sizes were observed in the uneven ancestry proportion when the causal variant MAF was lower in EUR than in EAS or AFR (S7 and S8 Tables).

#### Coverage is not comparable across all approaches.

**Fig 5:**
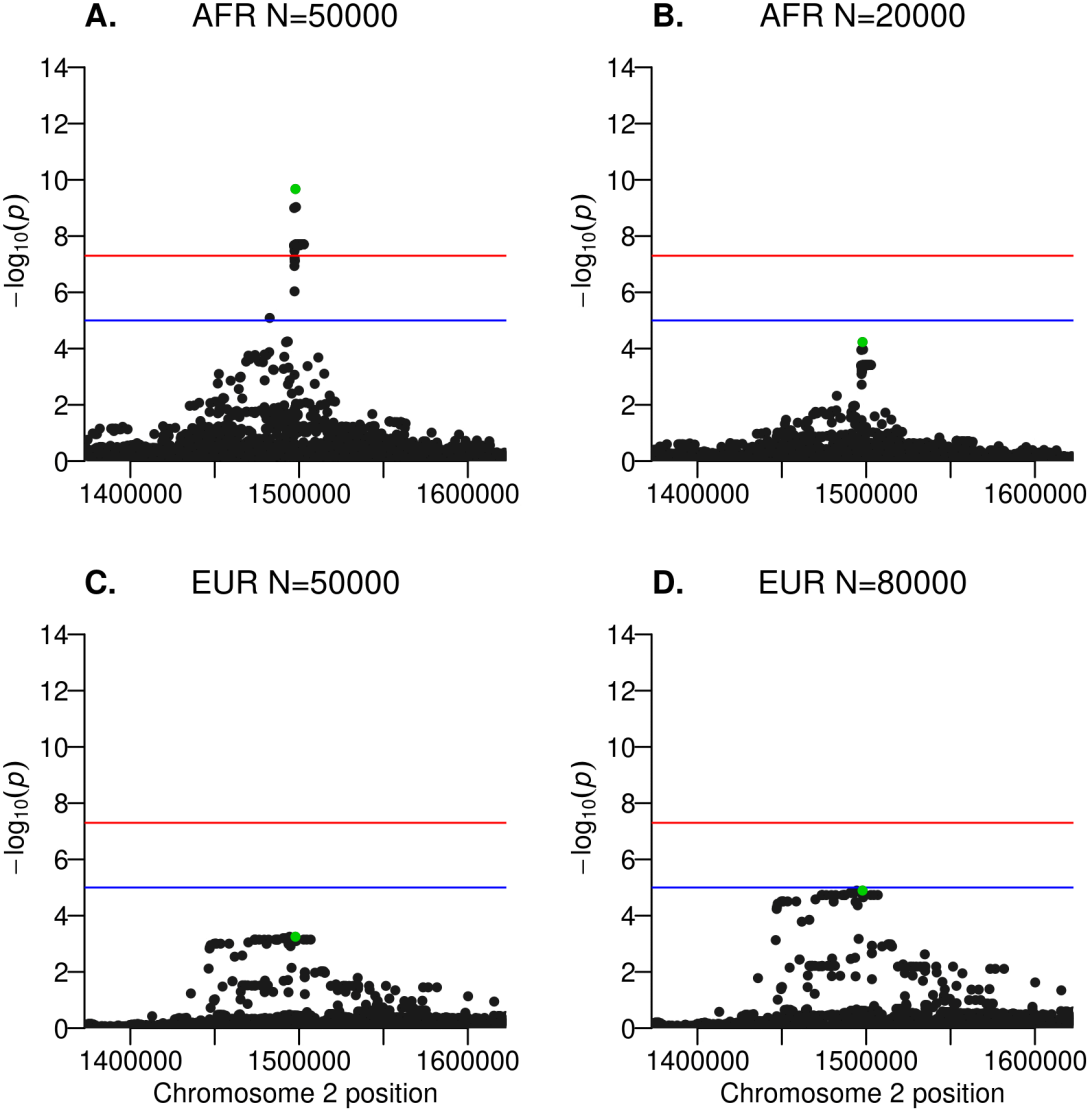
Manhattan plots of $-log_{10}$ p-values of expected z-scores calculated by simGWAS for a GWAS of a binary trait. The region is 1372831-1622831 on chromosome 2 (GRCh37), centered around causal variant rs10189329:1497831:G:A. The MAF of the causal variant is 0.05 and 0.25 in the EUR and AFR ancestries, respectively. A 1:4 case-control ratio was specified for both ancestries. **A**, **B** show AFR only p-values, with $N_{AFR}=50000,20000$, respectively. **C**, **D** show EUR only p-values with $N_{EUR}=50000,80000$, respectively.

Coverage is the proportion of simulation iterations where a colocalization credible set was constructed such that it included the colocalized variant. For SuSiEx based approaches, no credible sets are constructed if no colocalized variant is identified. In the simulation study of the region, chr2:1372831–162283 (GRCh37), coverage is comparable across all four approaches under the 50/50 EUR:AFR scenario. However, under the 80/20 EUR:AFR setting, SuSiEx based methods struggled to identify any colocalized variants because fine-mapping by SuSiEx often could not identify any causal variants for the GWAS and/or the eQTL trait. The causal variant MAF was high in AFR, but low in EUR. A smaller sample size in the AFR population decreased the power to detect the true causal variant, as shown in Fig 5A and 5B. The increased EUR sample size under the 80/20 EUR:AFR setting could not make up for the decreased power from a smaller AFR sample size, as shown in Fig 5C and 5D.

Under the 80/20 EUR:AFR scenario, the SuSiEx based approaches could not identify any colocalized variants, and no credible sets were constructed for 36 out of 100 iterations, resulting in the lower coverage shown in Fig 4B. However, when colocalization credible sets were constructed by the SuSiEx based approaches, they included the true colocalized variant 97% of the time. MsCAVIAR based approaches constructed credible sets for all 100 simulation iterations over this region, all included the true colocalized variant.

Across all 48 loci simulated to have one colocalized variant, the four approaches often reported comparable coverage to one another within each ancestry proportion (S3, S7 and S11 Figs). When the power to detect the causal variant is low, such as in the simulation setting where the GWAS summary statistics were simulated for the EUR and AFR ancestries based on a total sample size of *N* = 50,000, instead of *N* = 100,000, SuSiEx may struggle to identify any causal variants in the fine-mapping stage, so SuSiEx based approaches may not identify any colocalized variants (S4 Table).

#### Conditional variant level CLPP are comparable across all approaches.

cvCLPP is the probability that the variant is colocalized, given that the locus is colocalized. The cvCLPP of the true causal variant is of interest, it reflects the prioritization performance of the approach. In the simulation study over the region, chr2:1372831–162283 (GRCh37), all approaches report comparable cvCLPPs within each ancestry proportion (Fig 4C). This result is also observed in other loci simulated to have one colocalized variant (S4, S8 and S12 Figs). Between the ancestry proportions, the true causal variant in this locus is more strongly prioritized by all four approaches in the 50/50 EUR:AFR setting compared to the 80/20 EUR:AFR setting, keeping the total sample size fixed at *N* = 100,000 (Fig 4C). In 47 out of 48 of the single colocalized variant loci, the median cvCLPP reported by all four approaches under the uneven ancestry proportion setting was less than or equal to the cvCLPP reported under the even ancestry proportion setting, keeping the total sample size fixed at *N* = 100,000.

#### Locus level CLPP is low in eCAVIAR-based approaches.

lCLPP is the probability of colocalization within the locus. In the simulation study over the region, chr2:1372831–162283 (GRCh37), the eCAVIAR-based approaches report low lCLPPs, compared to the coloc-based approaches (Fig 4D). This is true for both the 50/50 EUR:AFR setting, and the 80/20 EUR:AFR setting. In both settings, coloc_SuSiEx and coloc_MsCAVIAR report median lCLPPs near 1.00, suggesting that the locus is colocalized. In both settings, the median lCLPPs reported by eMsCAVIAR and eCAVIAR_SuSiEx are less than 0.50. These results do not support colocalization within the locus.

In all 48 loci simulated to have one colocalized variant, both coloc-based approaches report median lCLPPs near 1.00 under both the balanced and unbalanced ancestry proportion settings where total *N* = 100,000. The median lCLPPs reported by the eCAVIAR-based approaches were less than 0.50 for 33 out of 48 loci under the 50/50 EUR:AFR scenario, and for 37 out of 48 loci under the 80/20 EUR:AFR setting (S5, S9 and S13 Figs).

### More than one causal variant

#### Differences in SuSiE and CAVIAR credible set definitions have implications in the two causal variant setting.

The performance of the approaches is investigated on four loci, each loci having two colocalized variants shared between traits. In the two causal variant scenario, the colocalization credible sets from the four approaches are not all comparable due to differences in the definition of a credible set between SuSiEx and MsCAVIAR. MsCAVIAR aims to construct a credible set containing all causal variants with at least γ probability, where γ is the user-specified coverage level. SuSiEx aims to construct a credible set containing at least one causal variant with at least γ probability, it is possible to return multiple credible sets in a multiple causal variant scenario. Thus, only the outputs between coloc_SuSiEx and eCAVIAR_SuSiEx are comparable, and only the results between eMsCAVIAR and coloc_MsCAVIAR are comparable.

When fine-mapping a trait over a region with 2 causal variants, SuSiEx returns 2 credible sets, each set capturing one causal variant. For the colocalization analysis of two traits (each trait having two signals in the locus), there are four colocalization comparisons under consideration by coloc_SuSiEx and eCAVIAR_SuSiEx. Of these four, two of these signals should return a high lCLPP (i.e., lCLPP > 0.50). The colocalization credible set for these two signals should each contain one of the two colocalized variants.

The fine-mapping method, MsCAVIAR aims to return one credible set for each trait, each set containing both causal variants. coloc or eCAVIAR would subsequently perform just one colocalization test. Both eMsCAVIAR and coloc_MsCAVIAR should construct one colocalization credible set with both colocalized variants included.

Colocalization analysis outputs differ between the SuSiEx and MsCAVIAR based methods. However, for SuSiEx, we can obtain a quantity that summarizes the overall lCLPP across the multiple credible sets by taking: $1-\prod_{k=1}^{CSpairs}(1-lCLPP)$, where $CS_{pairs}$ is the total number of fine-mapping credible set pairs to investigate for colocalized signals. This can be compared with the lCLPPs obtained by the MsCAVIAR based methods.

#### coloc_SuSiEx is the preferred approach for analysis of loci with multiple causal variants.

For all four regions with two colocalized variants, coloc_SuSiEx more frequently identified colocalization within the locus compared to all other approaches, followed by eCAVIAR_SuSiEx, eMsCAVIAR and coloc_MsCAVIAR (S9 Table). When comparing the two SuSiEx based approaches, coloc_SuSiEx more frequently returned two signals with high lCLPP compared to eCAVIAR_SuSiEx (S14–S17 Figs). For three of the four regions, eCAVIAR_SuSiEx identified at most one signal with a high lCLPP > 0.5 in all iterations (S14–S16 Figs). For one region it identified two signals with a high lCLPP in 40 out of 100 iterations (S17 Fig).

MsCAVIAR has a high computational cost. As a result, for eMsCAVIAR and coloc_MsCAVIAR, only 1 of the 4 regions completed all 100 iterations of colocalization analysis in the allotted time frame of 12 hours. For all regions, eMsCAVIAR reported very low lCLPPs. However, nearly all eMsCAVIAR credible sets (generated for the iterations that ran to completion) contained both causal variants (S9 Table). Since MsCAVIAR does not separate out signals into distinct credible sets, its outputs are incompatible with coloc for the colocalization analysis of loci with multiple causal variants. coloc_MsCAVIAR was investigated under the two causal variant scenario to verify the practical implications of this theoretical incompatibility. None of the regions reported any signal with a lCLPP > 0.5, all regions reported a mean locus level PP.H0 > 0.90 (S9 Table). This is the probability corresponding to hypothesis H0, that there is no association in the locus with either trait.

### No causal variants

#### All four approaches report no colocalization when the causal variants are distinct.

To evaluate the performance of the approaches under the absence of a true colocalized signal, the multi-ancestry colocalization approaches were used to analyze 2 traits that do not share a causal variant at the loci under investigation. All four approaches returned a mean lCLPP <0.05. In addition to the lCLPP (which is referred to as PP.H4 in coloc), the coloc based approaches also return PP.H3, the posterior probability for the hypothesis of distinct causal variants associated with each trait. coloc_SuSiEx reported a mean PP.H3 > 0.94 for all four loci. This is expected as the traits were simulated to be associated with distinct causal variants at the same locus. For 3 of the 4 regions, coloc_MsCAVIAR reported mean PP.H3 > 0.50. These results are also shown in S18 and S19 Figs.

### Simulation results summary

**Fig 6:**
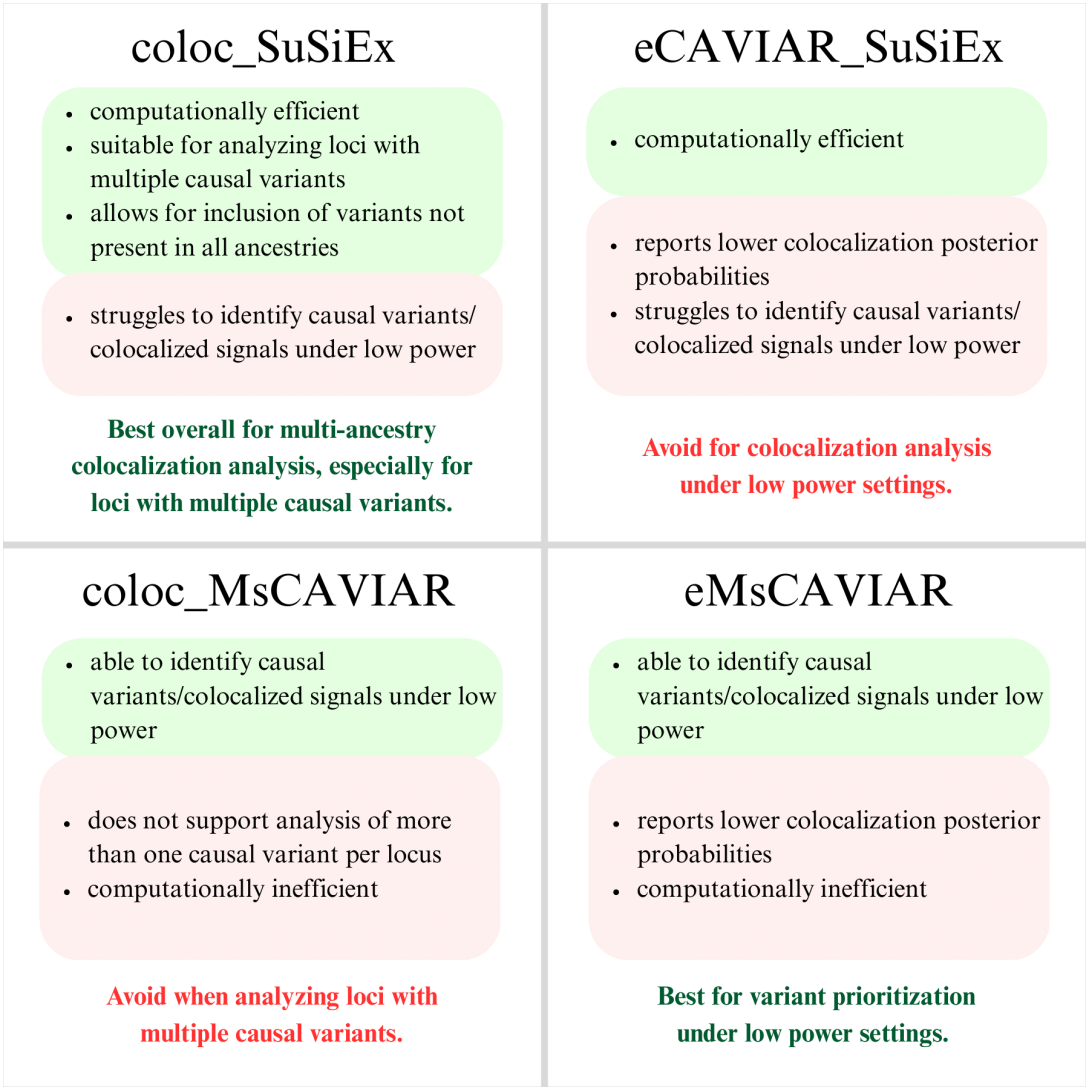
Comparison of strengths and limitations of the four proposed multi-ancestry colocalization approaches: coloc_SuSiEx, eCAVIAR_SuSiEx, coloc_MsCAVIAR and eMsCAVIAR.

The results from the simulation studies highlight the advantages and shortcomings of the four approaches, which are summarized in Fig 6.

We recommend first using coloc_SuSiEx to perform the colocalization analysis, as it is suitable for analyzing loci with multiple causal variants, and it is the only approach that allows for the inclusion of variants not present in all ancestries. SuSiEx based approaches, coloc_SuSiEx and eCAVIAR_SuSiEx are more computationally efficient than the MsCAVIAR based approaches. Runtimes for 100 iterations of fine-mapping analysis of 250kb loci with 1 causal variant was approximately 4 minutes by SuSiEx and 2.5 hours by MsCAVIAR. Runtimes for 100 iterations of fine-mapping analysis of 100kb loci with 2 causal variants was approximately 2 minutes by SuSiEx, but the same set of analyses by MsCAVIAR could not be completed in 12 hours. All analyses were performed using a single core of an Intel(R) Xeon(R) Gold 6242 CPU at 2.80 GHz. SuSiEx required approximately 50 MB of peak memory, while MsCAVIAR required approximately 300 MB. If no credible sets can be identified by SuSiEx due to low power, colocalization analysis by MsCAVIAR based approaches can be explored. However, coloc_MsCAVIAR should be avoided for analyzing loci with multiple causal variants.

### Real data application

To investigate the applicability of the proposed multi-ancestry colocalization methods on real datasets, coloc_SuSiEx, eMsCAVIAR, eCAVIAR_SuSiEx, coloc_MsCAVIAR were applied to analyze multi-ancestry T2D GWAS data from Mahajan et al. and circulating plasma protein (pQTL) data from European individuals in the INTERVAL study [9,10]. Mahajan et al. previously used the coloc method to perform a similar analysis, taking MR-MEGA meta-analyzed multi-ancestry T2D GWAS results and European pQTL data from the INTERVAL study [9]. MR-MEGA is a meta-analysis method for detecting SNP association, while allowing for heterogeneity in SNP effect sizes across ancestries by incorporating axes of genetic variation [11]. SuSiEx and MsCAVIAR use ancestry stratified summary statistics to perform multi-ancestry fine-mapping, explicitly modeling LD while allowing for effect size heterogeneity.

Results from colocalization analysis by coloc_SuSiEx, eMsCAVIAR, eCAVIAR_SuSiEx, coloc_MsCAVIAR are presented in S11–S14 Tables. Six loci (*PGM1, SLCO6A1-PAM, ABO, DLK1-MEG3, BCAR1, TOMM40-APOE-GIPR*) were investigated for colocalization analysis. Mahajan et al. previously reported one colocalized signal per region, except for *SLCO6A1-PAM* which had two putative colocalized variants, for a total of 7 colocalized signals. No approach was able to match exactly the results reported in [9]. coloc_SuSiEx assigned an lCLPP > 0.90 to 5 of the colocalization signals. coloc_SuSiEx identified a high posterior probability of colocalization between the T2D GWAS results at the *PGM1* loci and the PGM1 pQTL, at the *SLCO6A1-PAM* loci and the PAM pQTL, at the *BCAR1* loci and the CTRB1 pQTL, and at the *TOMM40-APOE-GIPR* loci and the APOE pQTL. These results indicate that the same causal signal drives both protein abundance and disease risk, and these findings are biologically consistent with known mechanisms, such as PGM1’s role in glucose metabolism [12], PAM’s role in activation of insulin-regulating peptides [13]. For the *DLK1-MEG3* locus, no coloc comparison returned a signal with lCLPP > 0.5. coloc_SuSiEx was unable to perform colocalization analysis for *ABO*, as SuSiEx did not return any credible sets for the pQTL dataset. eCAVIAR_SuSiEx returned a high lCLPP for the PAM locus, (CLPP = 1.00) and an lCLPP < 0.5 to all other regions, except for the *ABO* locus where SuSiEx did not return a credible set. Because MsCAVIAR constructs one credible set containing all causal variants for T2D and one credible set containing all causal variants for the pQTL dataset, there is only one coloc comparison to consider for each locus. Of the six loci analysed by coloc_MsCAVIAR, 3 loci (*APOE, ABO, PGM1*) returned an lCLPP > 0.5. eMsCAVIAR assigned a low lCLPP to all six loci, none exceeding 0.1.

## Discussion

This work explores the integration of multi-ancestry fine-mapping methods with single ancestry colocalization methods, producing four multi-ancestry colocalization approaches: coloc_SuSiEx, eMsCAVIAR, eCAVIAR_SuSiEx and coloc_MsCAVIAR. Simulation studies demonstrate the advantages and shortcomings of each approach. The SuSiEx-based approaches, coloc_SuSiEx and eCAVIAR_SuSiEx, are very efficient, which is a strength for multi-ancestry colocalization analysis of loci with multiple causal variants. In comparison, the MsCAVIAR-based approaches, coloc_MsCAVIAR and eMsCAVIAR, have higher computational burden but they are able to identify causal variants and colocalized signals even under low power settings where the SuSiEx based methods could not. Compared to the coloc-based approaches (coloc_SuSiEx, coloc_MsCAVIAR), the eCAVIAR-based approaches (eMsCAVIAR, eCAVIAR_SuSiEx) report lCLPPs that are too low. This is likely due to eCAVIAR’s simplifying assumption that the probability of a SNP being causal for one trait is independent of it being causal for another. Lastly, users should be aware that SuSiEx and MsCAVIAR define credible sets differently. Whereas SuSiEx aims to construct a credible set containing at least one causal variant, with the possibility of constructing multiple credible sets in a multiple causal variant scenario, MsCAVIAR aims to return one credible set containing all causal variants [2,4]. This difference in credible set construction results in incompatibility when integrating MsCAVIAR with coloc, which is observed when analyzing regions with more than one causal variant using coloc_MsCAVIAR. The coloc method was developed under the assumption of one causal variant per locus, it requires mutually exclusive posterior probabilities of a variant being causal, which SuSiEx can provide but MsCAVIAR does not. While coloc_MsCAVIAR is not recommended for analysis of loci with multiple colocalized variants, analysis of these loci using coloc_SuSiEx, ecAVIAR_SuSiEx and eMsCAVIAR is possible.

The applicability of the four multi-ancestry colocalization approaches on real data is explored by performing colocalization analysis on multi-ancestry T2D GWAS data from Mahajan et al. and pQTL datasets from European individuals in the INTERVAL study [9,10]. These results are compared with the colocalization results reported by Mahajan et al. in [9]. Whereas in this study, the T2D GWAS datasets were first analyzed by multi-ancestry fine-mapping methods, SuSiEx and MsCAVIAR, Mahajan et al. first meta-analyzed the multi-ancestry T2D GWAS data by MR-MEGA before colocalization analysis by coloc. Discrepancies in colocalization results can be explained by the different approaches, as well as the unavailability of the AFR and HIS T2D GWAS summary statistics. A limitation of the real data application in this report was that the pQTL dataset from the INTERVAL study included only individuals of European ancestry. A next step would be to apply the four proposed approaches to perform colocalization analysis on real data, both traits having multi-ancestry data available. Compared to single ancestry datasets, multi-ancestry datasets including individuals from diverse backgrounds are less common [14]. Nonetheless, as more initiatives bridge the gap in the availability of multi-ancestry datasets, the proposed multi-ancestry colocalization approaches will only become more applicable.

The four proposed approaches facilitate multi-ancestry colocalization analysis, but there are limitations. For eMsCAVIAR, coloc_MsCAVIAR, and eCAVIAR_SuSiEx, we note that variants not present in all ancestries have to be excluded from the analysis. While coloc_SuSiEx allows for variants missing in one ancestry, the assumption that the causal variants are shared across ancestries remains for all approaches. If this assumption must be relaxed, we recommend exploring the integration of colocalization methods with other fine-mapping approaches, like MESuSiE, which can explicitly handle ancestry specific causal variants [15]. Integration with recent methods like MultiSuSiE among others can also be explored [16,17].

For all multi-ancestry colocalization approaches, it is recommended to use in-sample LD as mismatch between the GWAS/eQTL sample and the reference panel may lead to spurious causal signals being identified by the fine-mapping approaches, which will be carried forward into the colocalization analysis [2]. Additionally, the quality of the data provided to these approaches can be another limitation, as it is assumed that the shared causal variants have been captured in the data. Fine-mapping results may also be miscalibrated when applied to GWAS results obtained from meta-analyzing heterogeneous cohorts [18].

Although the proposed approaches provide a workflow for multi-ancestry colocalization analysis, they all require input summary statistics that are stratified by ancestry, instead of multi-ancestry summary statistics. Methods that do not require ancestry stratification may benefit from increased power because of larger sample sizes as well as the inclusion of individuals that do not belong to a single ancestry group. However, summary statistics based multi-ancestry methods are challenging to implement because it may come at the cost of not leveraging LD information. As more multi-ancestry GWAS summary statistics become available, new methods that are truly multi-ancestry (and not ancestry stratified) will need to be developed.

## Methods

### Multi-ancestry colocalization approaches

Here, we introduce multi-ancestry colocalization approaches by integrating multi-ancestry fine-mapping methods SuSiEx and MsCAVIAR with single ancestry colocalization methods, coloc and eCAVIAR. The four proposed approaches are: coloc_SuSiEx, eMsCAVIAR, coloc_MsCAVIAR and eCAVIAR_SuSiEx.

#### coloc_SuSiEx.

coloc_SuSiEx first performs fine-mapping by SuSiEx and subsequently gives its output to coloc for analysis. Ancestry stratified summary statistics for trait 1 from ancestry 1 and ancestry 2 would be given as input to SuSiEx for multi-ancestry fine-mapping. Ancestry stratified summary statistics for trait 2 from ancestry 1 and ancestry 2 would be fine-mapped separately using SuSiEx. For each analysis, SuSiEx returns ancestry specific log Bayes factors at the variant level for each credible set identified. The following expression gives the Bayes factor for variant *j* in ancestry *i*, for *i* = 1,2,


$$\begin{array}{c} BF_{ij}=\frac{P(y_{i}|M_{1})}{P(y_{i}|M_{0})}\;. \end{array}$$ (1)

The Bayes factor compares how well the two models, *M*_1_ and *M*_0_ can explain $y_{i}$, the observed phenotypes for ancestry *i*. *M*_1_ is the model where the effect size of variant *j* in ancestry *i* is non-zero, and *M*_0_ is the null model of no causal variants.

The proposed multi-ancestry colocalization approach coloc_SuSiEx provides to coloc a combined log Bayes factor across two ancestries, as follows:


$$\begin{array}{cc} BF_{j} & =\frac{P(y_{1},y_{2}|M_{1})}{P(y_{1},y_{2}|M_{0})} \end{array}$$ (2)

$$\begin{array}{cc} & =\frac{P(y_{1}|M_{1})}{P(y_{1}|M_{0})}\times \frac{P(y_{2}|M_{1})}{P(y_{2}|M_{0})} \end{array}$$ (3)

$$\begin{array}{cc} & =BF_{1j}\times BF_{2j}\;, \end{array}$$ (4)

where the decomposition of the combined Bayes factor into the product of the ancestry specific Bayes factors can be justified as the data *y*_1_ and *y*_2_ come from different ancestries and are thus independent. The models being compared, *M*_1_ and *M*_0_ are consistent across the ancestries. In the R package **coloc**, the coloc.bf_bf() function colocalizes two datasets/traits that are represented by log Bayes factors, so the outputs of SuSiEx are provided to coloc. coloc_SuSiEx outputs the lCLPP, which is the posterior probability of *H*_4_ being true for the locus. cvCLPPs are also reported, these are the posterior probabilities that the variant is causal given that *H*4 is true. In regions where multiple causal variants are identified, coloc_SuSiEx outputs an lCLPP for each colocalization signal analyzed.

#### eMsCAVIAR.

The second approach we propose for multi-ancestry colocalization is named eMsCAVIAR. eMsCAVIAR follows a similar two-step process whereby multi-ancestry fine-mapping is first performed using MsCAVIAR, and a subsequent colocalization analysis follows using the eCAVIAR framework. MsCAVIAR aims to identify the smallest causal set, which is the set that contains all causal variants (though it may contain non-causal variants, too) [4]. Under the assumption that causal variants are shared across ancestries, MsCAVIAR outputs one causal set and the probability that each variant is contained in it. Ancestry stratified summary statistics are provided to MsCAVIAR for multi-ancestry fine-mapping of trait 1. This is repeated for trait 2. eMsCAVIAR subsequently takes the posterior probabilities of a SNP being included in the causal set from MsCAVIAR for trait 1 and trait 2 and multiplies them to obtain the variant level CLPP (vCLPP), following the eCAVIAR method [7]. The eCAVIAR assumption suggests that the probability of a SNP being causal for one trait is independent of it being causal for another [3]. The vCLPP can then be calculated by multiplying its causal posterior probabilities. Since causal posterior probabilities are not available from the MsCAVIAR output, the posterior probability of each variant to be included in the causal set is used as a proxy. In eMsCAVIAR, the vCLPP quantifies how likely a given variant is causal for both traits across all ancestries. The vCLPP is represented by $P(c_{j}^{\left(1\right)}=1,c_{j}^{\left(2\right)}=1|{\hat{s}}_{1}^{\left(1\right)},{\hat{s}}_{1}^{\left(2\right)},{\hat{s}}_{2}^{\left(1\right)},{\hat{s}}_{2}^{\left(2\right)})$, where ${\hat{s}}_{i}^{\left(1\right)}$ for *i* = 1,2 is the trait 1 summary statistics from ancestry *i*, ${\hat{s}}_{i}^{\left(2\right)}$ is the trait 2 summary statistics from ancestry *i*, and $c_{j}^{\left(1\right)}$, $c_{j}^{\left(2\right)}$ are the causal status of the $j^{th}$ SNP in the GWAS and eQTL study, respectively. Under the eCAVIAR assumption, the vCLPP can be calculated as follows:


$$\begin{array}{c} P(c_{j}^{\left(1\right)}=1,c_{j}^{\left(2\right)}=1|{\hat{s}}_{1}^{\left(1\right)},{\hat{s}}_{2}^{\left(1\right)},{\hat{s}}_{1}^{\left(2\right)},{\hat{s}}_{2}^{\left(2\right)})=P(c_{j}^{\left(1\right)}=1|{\hat{s}}_{1}^{\left(1\right)},{\hat{s}}_{2}^{\left(1\right)})\times P(c_{j}^{\left(2\right)}=1|{\hat{s}}_{1}^{\left(2\right)},{\hat{s}}_{2}^{\left(2\right)})\;. \end{array}$$ (5)

Taking the sum of all vCLPPs in the locus gives the lCLPP. cvCLPPs can be taken as the vCLPPs divided by the lCLPP.

#### coloc_MsCAVIAR.

coloc_MsCAVIAR performs multi-ancestry fine-mapping for trait 1 and then trait 2 using MsCAVIAR, and subsequently performs colocalization analysis by coloc. The coloc method requires variant level log Bayes factors for each trait as input. For each trait, these Bayes factors compare *M*_1_, the model where the variant is truly causal, to *M*_0_, the null model of no causal variants. This is shown in (2). MsCAVIAR returns the posterior probability that each variant is included in the causal set, it does not consider *M*_0_, the null model of no causal variants. With this limitation, Bayes factors comparing the inclusion (model $M_{j}$) or exclusion (model *M*_-*j*_) of a variant in a causal set were used instead, allowing for an approximate integration of MsCAVIAR outputs with coloc. The Bayes factor for trait *k*, for *k* = 1,2 and variant *j* can be obtained from the MsCAVIAR posterior probabilities in the following way:


$$\begin{array}{cc} BF_{jk} & =\frac{P(y_{1},y_{2}|M_{j})}{P(y_{1},y_{2}|M_{-j})} \end{array}$$ (6)

$$\begin{array}{cc} & =\frac{P(M_{-j})}{P(M_{-j}|y_{1},y_{2})}\times \frac{P(M_{j}|y_{1},y_{2})}{P(M_{j})} \end{array}$$ (7)

$$\begin{array}{cc} & =\frac{1-P(M_{j})}{P(M_{j})}\times \frac{P(M_{1}|y_{1},y_{2})}{1-P(M_{j}|y_{1},y_{2})}\;, \end{array}$$ (8)

where $y_{i}$ for *i* = 1,2 are the observed phenotypes for ancestry *i*, $M_{j}$ is the model where variant *j* is causal, and *M*_-*j*_ is the model where variant *j* is not causal. coloc_MsCAVIAR will return the lCLPP, and cvCLPPs.

#### eCAVIAR_SuSiEx.

eCAVIAR_SuSiEx first performs fine-mapping by SuSiEx. This step returns credible sets and the posterior inclusion probabilities (PIPs) for each variant to belong in each credible set. This output is provided to eCAVIAR for colocalization analysis. The vCLPPS from eCAVIAR_SuSiEx come from multiplying the SuSiEx PIPs for trait 1 by the SuSiEx PIPs for trait 2, following the eCAVIAR method. Taking the sum of all vCLPPs in the locus gives the lCLPP. cvCLPPs can be taken as the vCLPPs divided by the lCLPP.

### Simulation strategy

The performance of the proposed multi-ancestry colocalization approaches are investigated under different simulation settings. 1000 Genomes phase 3 data [19], phased by IMPUTE2 [20] provided haplotypes for EUR, AFR and EAS samples. GWAS summary statistics for a binary trait were simulated using simGWAS [21], with LD and MAFs calculated from the reference haplotypes. Summary statistics for quantitative traits were simulated using GWASBrewer [22], also using the reference haplotypes to calculate the LD and MAFs. In depth details of all simulation settings can be found in S1–S10 Tables. Table 2 gives a summary of the simulation settings across all studies.

GWAS and eQTL datasets were simulated such that the causal variant OR and/or BETA was as specified in Table 2, with 100000 as the total sample size across all ancestries. A 1:4 case control ratio was specified for the binary traits. The locus was specified to be 250 kbp centered around the causal variant for all simulation studies, excepting the two causal variant setting, where the locus size was computationally inefficient to analyze for the MsCAVIAR-based approaches, and 100 kbp was used instead. For each region, 100 summary statistics datasets for trait 1 were simulated and 100 datasets for trait 2 were simulated so that 100 iterations of multi-ancestry colocalization analysis could be performed. Ancestry specific summary statistics for trait 1 were input to SuSiEx or MsCAVIAR for multi-ancestry fine-mapping. This was repeated for trait 2. The multi-ancestry fine-mapping results for trait 1 and trait 2 were then input to coloc or eCAVIAR for colocalization analysis.

**Table 2:** Summary of simulation study settings.

Setting	OR	BETA	N_EUR	N_AFR	N_EAS	Locus Size (kbp)	# Loci
Similar MAF, 50/50 EUR:AFR	1.11, 1.12, 1.24	NA	50000	50000	NA	250	12
Similar MAF, 80/20 EUR:AFR	1.11, 1.12, 1.24	NA	80000	20000	NA	250	12
Similar MAF, 50/50 EUR: AFR	1.11, 1.12, 1.24	NA	25000	25000	NA	250	12
Similar MAF, 50/50 EUR: AFR	1.15, 1.17, 1.35	NA	25000	25000	NA	250	12
Different MAF, 50/50 EUR:AFR	1.12	0.05	50000	50000	NA	250	12
Diferent MAF, 80/20 EUR:AFR	1.12	0.05	80000	20000	NA	250	12
3 Ancestries, 33/33/33 EUR:EAS:AFR	1.12	0.05	33334	33333	33333	250	24
3 Ancestries, 75/15/10 EUR:EAS:AFR	1.12	0.05	75000	10000	15000	250	24
2 Causal Variants, 50/50 EUR:AFR	1.12	0.05	50000	50000	NA	100	4
Distinct Causal Variants, 50/50 EUR:AFR	1.12	0.05	50000	50000	NA	250	4

In the Similar MAF scenarios, colocalization was performed between two binary traits. All other scenarios used one binary and one quantitative trait. For the Similar MAF scenarios, three odds ratios were chosen such that the MAF of the causal variant varied between 0.05 to 0.50 while maintaining 80% power. In all other scenarios, the causal variant MAF ranged from 0.05 to 0.50 but the effect sizes were fixed, resulting in varying power across simulations. The proportion of cases in all binary-trait simulations was fixed at 0.20. “# Loci” indicates the number of simulated regions for which colocalization analysis was performed under that scenario.

#### One causal variant, similar MAF between EUR and AFR, 50/50 and 80/20 EUR:AFR.

The performance of the proposed multi-ancestry colocalization approaches were studied first under the most basic simulation setting, involving the colocalization of two binary traits that share the same causal variant, whose MAF is similar between the EUR and AFR ancestries. Within this setting, we consider two ancestry sample compositions: balanced, where $N_{EUR}$ = $N_{AFR}$ = 50000, and unbalanced, where $N_{EUR}$ = 80000 and $N_{AFR}$ = 20000. These sample sizes and compositions are applied to simulate summary statistics for both GWAS traits. Twelve causal variants were randomly selected from all variants that had similar MAFs across both EUR and AFR ancestries. Twelve regions were defined by these selected causal variants. The regions had varying LD patterns – low LD in both ancestries, high LD in both ancestries, low LD in EUR but high in AFR, and high LD in EUR but low in AFR. Causal variant MAFs were either 0.50, 0.25, or 0.05 in both the EUR and AFR ancestries. Using QUANTO [23] to compute the expected power of detecting an association, the odds ratio for the causal variant was specified to be one of 1.11 (MAF = 0.5), 1.12 (MAF = 0.25) or 1.24 (MAF = 0.05) such that the power at significance level $5\times 10^{-8}$ was 80% when $N_{EUR}=N_{AFR}=50000$. In the 80/20 EUR:AFR scenario, the power to detect the causal variant in the EUR and AFR ancestries is 99% and 7%, respectively.

#### One causal variant, similar MAF between EUR and AFR, 50/50 EUR:AFR, reduced sample size.

The performance of the proposed multi-ancestry colocalization approaches are also studied under reduced GWAS and eQTL sample sizes. Using the same 12 loci as described in the previous section, we simulated GWAS and eQTL summary statistics based on $N_{EUR}=N_{AFR}=25000$. We first consider the scenario where the effect sizes are specified exactly as described in the previous section. The power at significance level $5\times 10^{-8}$ was reduced from 80% to 16%. We consider a second scenario where the effect sizes have changed, such that the power at significance level $5\times 10^{-8}$ remains at 80%, despite the decreased total sample size from *N* = 100000 to *N* = 50000.

#### One causal variant, different MAF in EUR and AFR, 50/50 and 80/20 EUR:AFR.

The performance of the proposed multi-ancestry colocalization methods is evaluated when the MAFs of the causal variant vary across the ancestries. We simulate one binary GWAS and one continuous eQTL trait that share the same causal variant, whose MAF is different across the EUR and AFR ancestries. Within this setting, we again consider the balanced ancestry composition where $N_{EUR}=N_{AFR}=50000$ for the GWAS and eQTL trait, and the unbalanced ancestry composition where $N_{EUR}=80000,N_{AFR}=20000$ for both traits. The odds ratio and the effect size of the causal variant was set to OR = 1.12 and BETA = 0.05, such that the expected power at significance level $5\times 10^{-8}$ is 80% when the MAF of the causal variant is 0.25, and $N_{EUR}=N_{AFR}=50000$. The power to detect the causal variant in each ancestry depends on the MAF and sample size, and increases as the sample size or MAF increases, or decreases as the sample size or MAF decreases. The detailed power settings are provided in S5 and S6 Tables. We consider causal variants with EUR MAF = 0.05 with AFR MAFs = 0.25 or 0.50; EUR MAF = 0.25 with AFR MAFs = 0.05 or 0.50; EUR MAF = 0.50 with AFR MAFs = 0.05 or 0.25. Twelve causal variants satisfying these MAF criteria were randomly selected. Twelve loci were simulated and analyzed by all four approaches.

#### One causal variant, 33/33/33 and 75/15/10 EUR:EAS:AFR.

The performance of the four methods is evaluated on GWAS and eQTL datasets including three ancestries: EUR, AFR and EAS. Again, we consider the balanced ancestry sample composition where $N_{EUR}$ = $N_{AFR}$ = $N_{EAS}=33333$ for both traits, and the unbalanced composition where $N_{EUR}=75000$, $N_{EAS}=15000$ and $N_{AFR}=10000$ for both traits. The OR and BETA of the causal variant were set to 1.12 and 0.05 respectively to be consistent with settings of the previous simulation study. Detailed power settings are available in S7 and S8 Tables. We consider causal variants with varied MAFs = 0.05, 0.25, 0.50 across the ancestries. Twenty four variants were randomly selected to be causal, and twenty four loci were defined around these variants and analyzed by all four approaches.

#### Two causal variants.

The performance of the proposed multi-ancestry colocalization approaches is evaluated on loci with 2 causal variants, both of which are shared between the binary and the quantitative trait. We consider only the balanced sample composition where $N_{EUR}=50000,N_{AFR}=50000$. Four pairs of causal variants were selected, such that the LD between them ranged from *r*^2^ of 0.06 to 0.88 in the EUR ancestry and 0.02 to 0.36 in the AFR ancestry. Each region was defined to be 100 kbp centered around the midpoint of the pair of causal variants.

#### Distinct causal variants.

Two traits with an association signal in the same locus can be explained by different causal variants. In this case, the traits are not colocalized at this locus. A simulation study of the scenario with two distinct causal variants not shared between the traits was performed to assess how often the multi-ancestry colocalization approaches detect colocalization when there is none. Four loci were selected such that the causal variant for trait 1 in the locus was distinct from the causal variant for trait 2. The LD between the two selected causal variants ranged from *r*^2^ of 0.06 to 0.37 in EUR and 0.06 to 0.48 in AFR. We consider only the balanced sample composition where $N_{EUR}=50000,N_{AFR}=50000$. Each region was defined to be 250 kbp centered around the midpoint of the 2 causal variants.

### Real data application

The proposed multi-ancestry colocalization methods were applied to multi-ancestry type 2 diabetes (T2D) GWAS summary statistics from Mahajan et al. [9], and EUR ancestry plasma protein QTL summary statistics from the INTERVAL study [10]. Mahajan et al. assembled T2D GWAS summary statistics from 122 studies, including individuals from five ancestry groups: EUR, AFR, EAS, SAS, and HIS. GWAS summary statistics from the EUR, EAS and SAS ancestries were available, with $N_{cases}=80154,56268,16540$, respectively, and $N_{controls}=853816,227155,32952$, respectively. The INTERVAL study investigated genetic associations with levels of nearly 3000 different plasma proteins in 3301 healthy individuals of EUR ancestry.

We considered only variants with MAF > 0.5% in at least one ancestry, available in the 1000G Phase 3 reference panel for analysis by coloc_SuSiEx. The other proposed approaches eMsCAVIAR, coloc_MsCAVIAR, and eCAVIAR_SuSiEx only include SNPs that are available in all ancestries and all traits for the analysis.

## Supporting information

S1 FigManhattan plots of the $-log_{10}$ p-values of expected z-scores calculated by GWASBrewer for a GWAS of a continuous trait (such as an eQTL).The region is 1372831–1622831 on chromosome 2 (GRCh37), centered around causal variant rs10189329:1497831:G:A. The MAF of the causal variant is 0.05 and 0.25 in the EUR and AFR ancestries, respectively. C), D) show EUR only p-values, with $N_{EUR}=50000,80000$, respectively. E), F) show AFR only p-values with $N_{AFR}=50000,20000$, respectively. SNP p-values from a fixed effects meta analysis over both ancestries are plotted in A) with $N_{EUR}=50000,N_{AFR}=50000$ and B) with $N_{EUR}=80000,N_{AFR}=20000$, respectively.(PDF)

S2 Fig Median credible set sizes returned by the multi-ancestry colocalization approaches for loci simulated under the one causal variant, similar MAF in EUR and AFR setting. Two ancestry proportions were considered: 50/50 and 80/20 EUR:AFR, for a total N = 100000 in each setting. 95% colocalization credible sets were constructed by ranking variants by conditional variant level CLPPs and summing until the cumulative CLPP exceeds 0.95.
(PDF)

S3 Fig Coverage reported by the multi-ancestry colocalization approaches for loci simulated under the one causal variant, similar MAF in EUR and AFR setting. Two ancestry proportions were considered: 50/50 and 80/20 EUR:AFR, for a total N = 100000 in each setting. Coverage is the proportion of iterations the causal variant was included in the 95% credible set out of 100 iterations.
(PDF)

S4 Fig Conditional variant level CLPP of the true colocalized variant reported by the multi-ancestry colocalization approaches for loci simulated under the one causal variant, similar MAF in EUR and AFR setting. Two ancestry proportions were considered: 50/50 and 80/20 EUR:AFR, for a total N = 100000 in each setting. Conditional variant level CLPP is the probability the variant is colocalized, given that the locus is colocalized.
(PDF)

S5 Fig Locus level CLPP reported by the multi-ancestry colocalization approaches for loci simulated under the one causal variant, similar MAF in EUR and AFR setting. Two ancestry proportions were considered: 50/50 and 80/20 EUR:AFR, for a total N = 100000 in each setting. Locus level CLPP is the probability that the locus is colocalized.
(PDF)

S6 Fig Median credible set sizes returned by the multi-ancestry colocalization approaches for loci simulated under the one causal variant, different MAF in EUR and AFR setting. Two ancestry proportions were considered: 50/50 and 80/20 EUR:AFR, for a total N = 100000 in each setting. 95% colocalization credible sets were constructed by ranking variants by conditional variant level CLPPs and summing until the cumulative CLPP exceeds 0.95.
(PDF)

S7 Fig Coverage reported by the multi-ancestry colocalization approaches for loci simulated under the one causal variant, different MAF in EUR and AFR setting. Two ancestry proportions were considered: 50/50 and 80/20 EUR:AFR, for a total N = 100000 in each setting. Coverage is the proportion of iterations the causal variant was included in the 95% credible set out of 100 iterations.
(PDF)

S8 Fig Conditional variant level CLPP of the true colocalized variant reported by the multi-ancestry colocalization approaches for loci simulated under the one causal variant, different MAF in EUR and AFR setting. Two ancestry proportions were considered: 50/50 and 80/20 EUR:AFR, for a total N = 100000 in each setting. Conditional variant level CLPP is the probability the variant is colocalized, given that the locus is colocalized.
(PDF)

S9 Fig Locus level CLPP reported by the multi-ancestry colocalization approaches for loci simulated under the one causal variant, different MAF in EUR and AFR setting. Two ancestry proportions were considered: 50/50 and 80/20 EUR:AFR, for a total N = 100000 in each setting. Locus level CLPP is the probability that the locus is colocalized.
(PDF)

S10 Fig Median credible set sizes returned by the multi-ancestry colocalization approaches for loci simulated under the one causal variant, multiple ancestries setting. Two ancestry proportions were considered: 33/33/33 and 75/15/10 EUR:EAS:AFR, for a total N = 100000 in each setting. 95% colocalization credible sets were constructed by ranking variants by conditional variant level CLPPs and summing until the cumulative CLPP exceeds 0.95.
(PDF)

S11 Fig Coverage reported by the multi-ancestry colocalization approaches for loci simulated under the one causal variant, multiple ancestries setting. Two ancestry proportions were considered: 33/33/33 and 75/15/10 EUR:EAS:AFR, for a total N = 100000 in each setting. Coverage is the proportion of iterations the causal variant was included in the 95% credible set out of 100 iterations.
(PDF)

S12 Fig Conditional variant level CLPP of the true colocalized variant reported by the multi-ancestry colocalization approaches for loci simulated under the one causal variant, multiple ancestries setting. Two ancestry proportions were considered: 33/33/33 and 75/15/10 EUR:EAS:AFR, for a total N = 100000 in each setting. Conditional variant level CLPP is the probability the variant is colocalized, given that the locus is colocalized.
(PDF)

S13 Fig Locus level CLPP reported by the multi-ancestry colocalization approaches for loci simulated under the one causal variant, multiple ancestries setting. Two ancestry proportions were considered: 33/33/33 and 75/15/10 EUR:EAS:AFR, for a total N = 100000 in each setting. Locus level CLPP is the probability that the locus is colocalized.
(PDF)

S14 Fig coloc_SuSiEx and eCAVIAR_SuSiEx results for the 2 causal variants, 2 ancestries setting. Multi-ancestry colocalization approaches were evaluated on chr2:191476292–191576292 (GRCh37), centered around causal variants rs10200534:191537142:C:A and, rs1023568:191515442:A:T by A. coloc_SuSiEx, B. eCAVIAR_SuSiEx. For coloc comparisons with a locus level CLPP > 0.5, a 95% colocalization credible set was constructed by ranking variants by conditional variant level CLPPs and summing until the cumulative CLPP exceeds 0.95.
(PDF)

S15 Fig coloc_SuSiEx and eCAVIAR_SuSiEx results for the 2 causal variants, 2 ancestries setting. Multi-ancestry colocalization approaches were evaluated on chr2:171130765–171230765 (GRCh37), centered around causal variants rs59441748:171185685:C:A and rs716427:171175844:A:G by A. coloc_SuSiEx, B. eCAVIAR_SuSiEx. For coloc comparisons with a locus level CLPP > 0.5, a 95% colocalization credible set was constructed by ranking variants by conditional variant level CLPPs and summing until the cumulative CLPP exceeds 0.95.
(PDF)

S16 Fig coloc_SuSiEx and eCAVIAR_SuSiEx results for the 2 causal variants, 2 ancestries setting. Multi-ancestry colocalization approaches were evaluated on 50/50 EUR:AFR datasets on chr2:17265320–17365320 (GRCh37), centered around causal variants rs71447280:17325432:A:G and rs60143916:17305207:G:A by A. coloc_SuSiEx, B. eCAVIAR_SuSiEx. For coloc comparisons with a locus level CLPP > 0.5, a 95% colocalization credible set was constructed by ranking variants by conditional variant level CLPPs and summing until the cumulative CLPP exceeds 0.95.
(PDF)

S17 Fig coloc_SuSiEx and eCAVIAR_SuSiEx results for the 2 causal variants, 2 ancestries setting. Multi-ancestry colocalization approaches were evaluated on 50/50 EUR:AFR datasets on chr2:67906038–68006038 (GRCh37), centered around causal variants rs142101372:67967864:A:C and rs1864572:67944212:T:C by A. coloc_SuSiEx, B. eCAVIAR_SuSiEx. For coloc comparisons with a locus level CLPP > 0.5, a 95% colocalization credible set was constructed by ranking variants by conditional variant level CLPPs and summing until the cumulative CLPP exceeds 0.95.
(PDF)

S18 Fig Locus level CLPP reported by the multi-ancestry colocalization approaches for loci simulated under the distinct causal variants setting. Multi-ancestry colocalization analysis approaches were applied to analyze EUR and AFR datasets on loci with distinct causal variants across traits, that is, on loci that are not colocalized across the traits under analysis. The locus level CLPP is the probability of colocalization within the locus.
(PDF)

S19 Fig PP.H3 reported by coloc_SuSiEx and coloc_MsCAVIAR for loci simulated under the distinct causal variants setting. Multi-ancestry colocalization analysis approaches were applied to analyze EUR and AFR datasets on loci with distinct causal variants across traits, that is, on loci that are not colocalized across the traits under analysis. PP.H3 is the posterior probability corresponding to coloc’s third hypothesis, where two traits have an association signal at the same locus, but the association signals are explained by different causal variants.
(PDF)

S1 Table Simulation study results for the setting: One causal variant, similar MAF, 50/50 EUR:AFR, total N = 100000. (XLSX)

S2 Table Simulation study results for the setting: One causal variant, similar MAF, 80/20 EUR:AFR, total N = 100000. (XLSX)

S3 Table Simulation study results for the setting: One causal variant, similar MAF, 50/50 EUR:AFR, total N = 50000. (XLSX)

S4 Table Simulation study results for the setting: One causal variant, similar MAF, 50/50 EUR:AFR, total N = 50000, reduced power. (XLSX)

S5 Table Simulation study results for the setting: One causal variant, different MAF, 50/50 EUR:AFR, total N = 100000. (XLSX)

S6 Table Simulation study results for the setting: One causal variant, different MAF, 80/20 EUR:AFR, total N = 100000. (XLSX)

S7 Table Simulation study results for the setting: One causal variant, 33/33/33 EUR:EAS:AFR, total N = 100000. (XLSX)

S8 Table Simulation study results for the setting: One causal variant, 75/15/10 EUR:EAS:AFR, total N = 100000. (XLSX)

S9 Table Simulation study results on the coloc_MsCAVIAR and eMsCAVIAR colocalization analysis of the 2 causal variant settings, 50/50 EUR:AFR, total N = 100000. (XLSX)

S10 Table Simulation study results for the negative control, distinct causal variant setting. (XLSX)

S11 Table Comparison of coloc_SuSiEx colocalization results with colocalization results from Mahajan et al. on multi-ancestry T2D GWAS data from the DIAMANTE Consortium with European pQTL data from the INTERVAL study. (XLSX)

S12 Table Comparison of coloc_MsCAVIAR colocalization results to the colocalization results from Mahajan et al. on multi-ancestry T2D GWAS data from the DIAMANTE Consortium with European pQTL data from the INTERVAL study. (XLSX)

S13 Table Comparison of eMsCAVIAR colocalization results with colocalization results from Mahajan et al. on multi-ancestry T2D GWAS data from the DIAMANTE Consortium with European pQTL data from the INTERVAL study. (XLSX)

S14 Table Comparison of eCAVIAR_SuSiEx colocalization results with colocalization results from Mahajan et al. on multi-ancestry T2D GWAS data from the DIAMANTE Consortium with European pQTL data from the INTERVAL study. (XLSX)
