# Supplementary figures and images for: Multi-ancestry colocalization approaches
Source: PLoS Genet. 2026 Jul 21;22(7):e1012221. doi: 10.1371/journal.pgen.1012221 (PMC13387578; doi:10.1371/journal.pgen.1012221)

**A**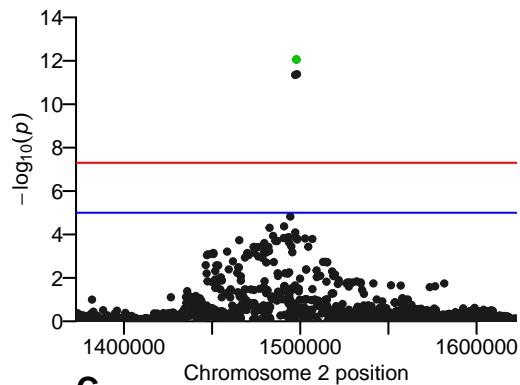**B**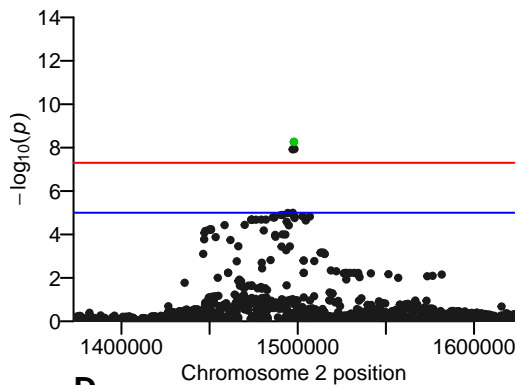**C**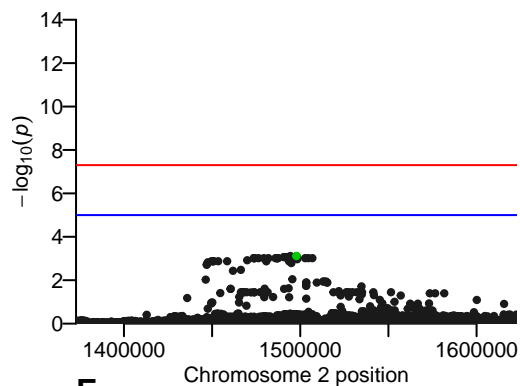**D**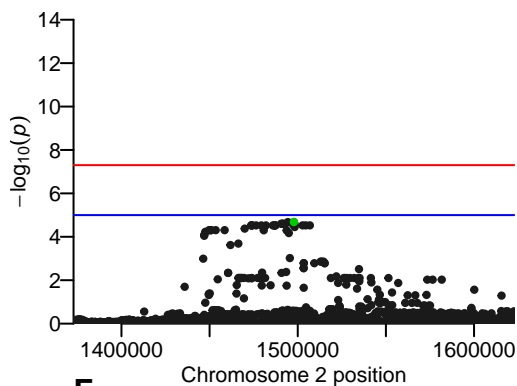**E**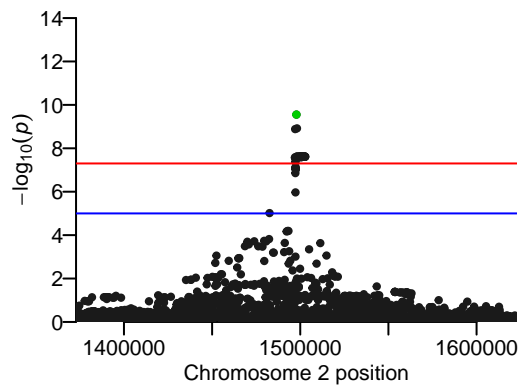**F**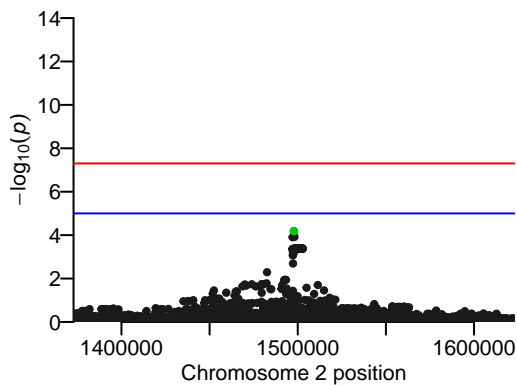

Supplement: S1 Fig — The region is 1372831–1622831 on chromosome 2 (GRCh37), centered around causal variant rs10189329:1497831:G:A. The MAF of the causal variant is 0.05 and 0.25 in the EUR and AFR ancestries, respectively. C), D) show EUR only p-values, with NEUR=50000,80000, respectively. E), F) show AFR only p-values with NAFR=50000,20000, respectively. SNP p-values from a fixed effects meta analysis over both ancestries are plotted in A) with NEUR=50000,NAFR=50000 and B) with NEUR=80000,NAFR=20000, respectively. (PDF) [file pgen.1012221.s001.pdf]

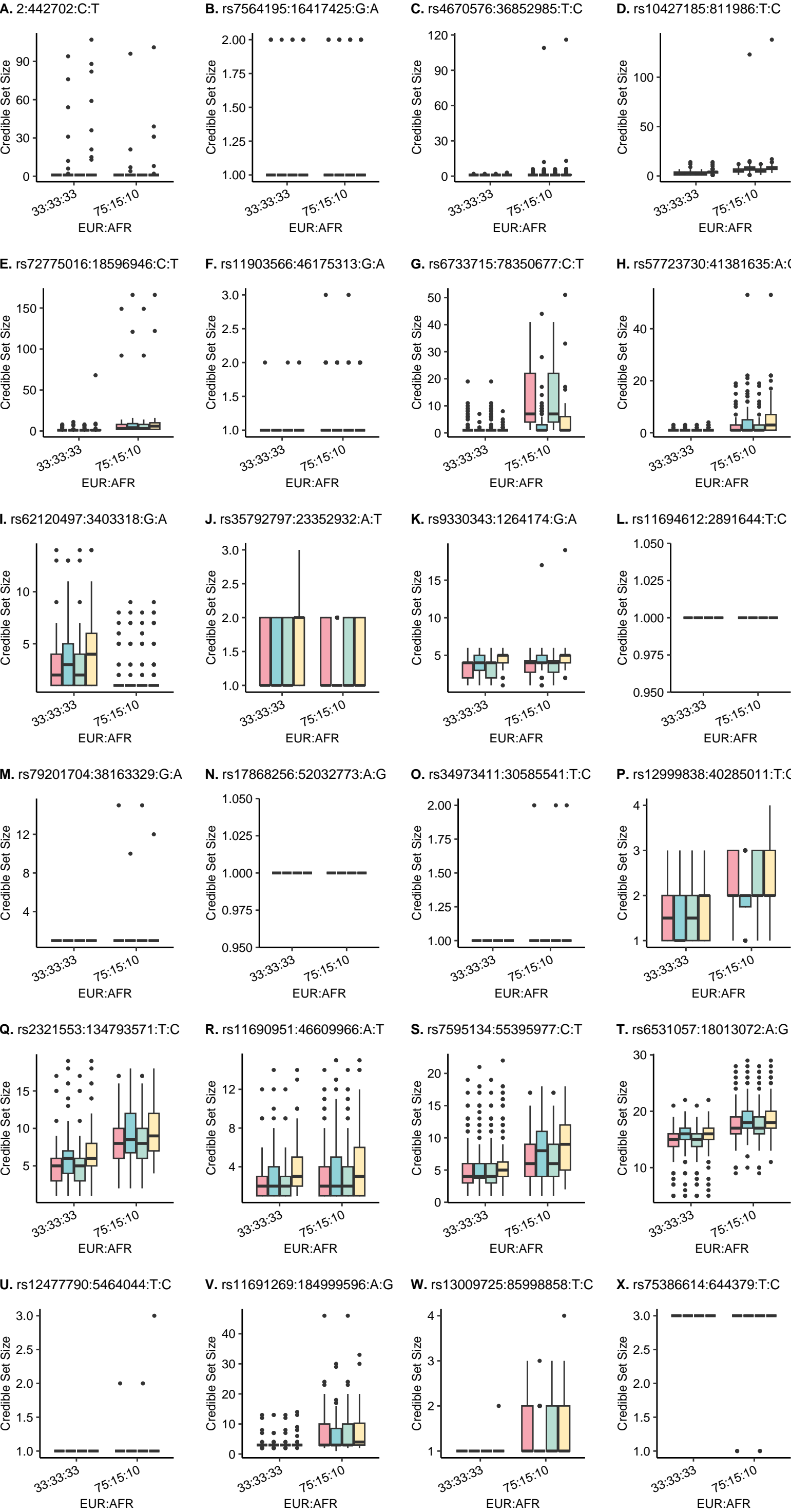

Supplement: S10 Fig — Two ancestry proportions were considered: 33/33/33 and 75/15/10 EUR:EAS:AFR, for a total N = 100000 in each setting. 95% colocalization credible sets were constructed by ranking variants by conditional variant level CLPPs and summing until the cumulative CLPP exceeds 0.95. (PDF) [file pgen.1012221.s010.pdf]

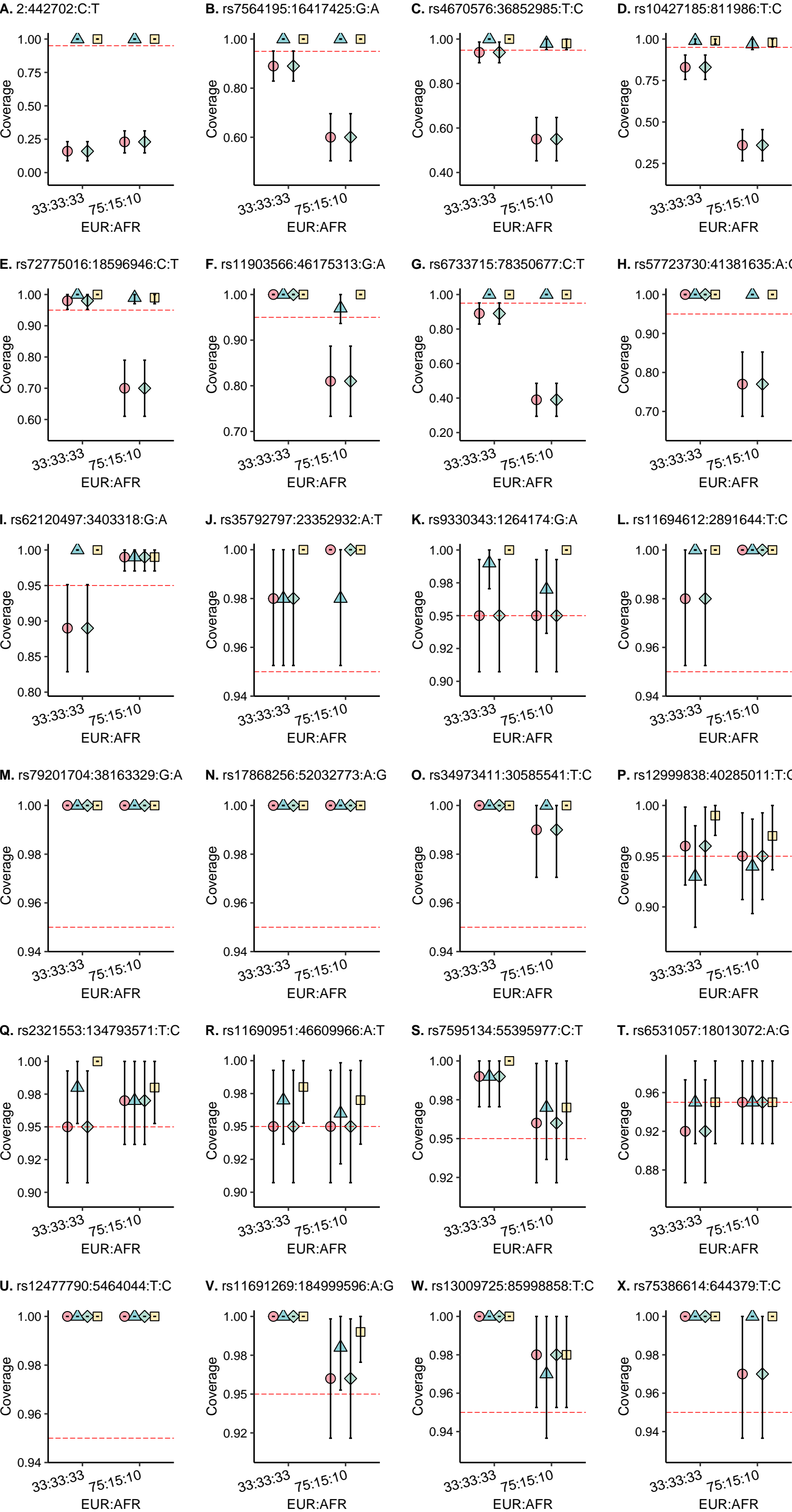

Supplement: S11 Fig — Two ancestry proportions were considered: 33/33/33 and 75/15/10 EUR:EAS:AFR, for a total N = 100000 in each setting. Coverage is the proportion of iterations the causal variant was included in the 95% credible set out of 100 iterations. (PDF) [file pgen.1012221.s011.pdf]

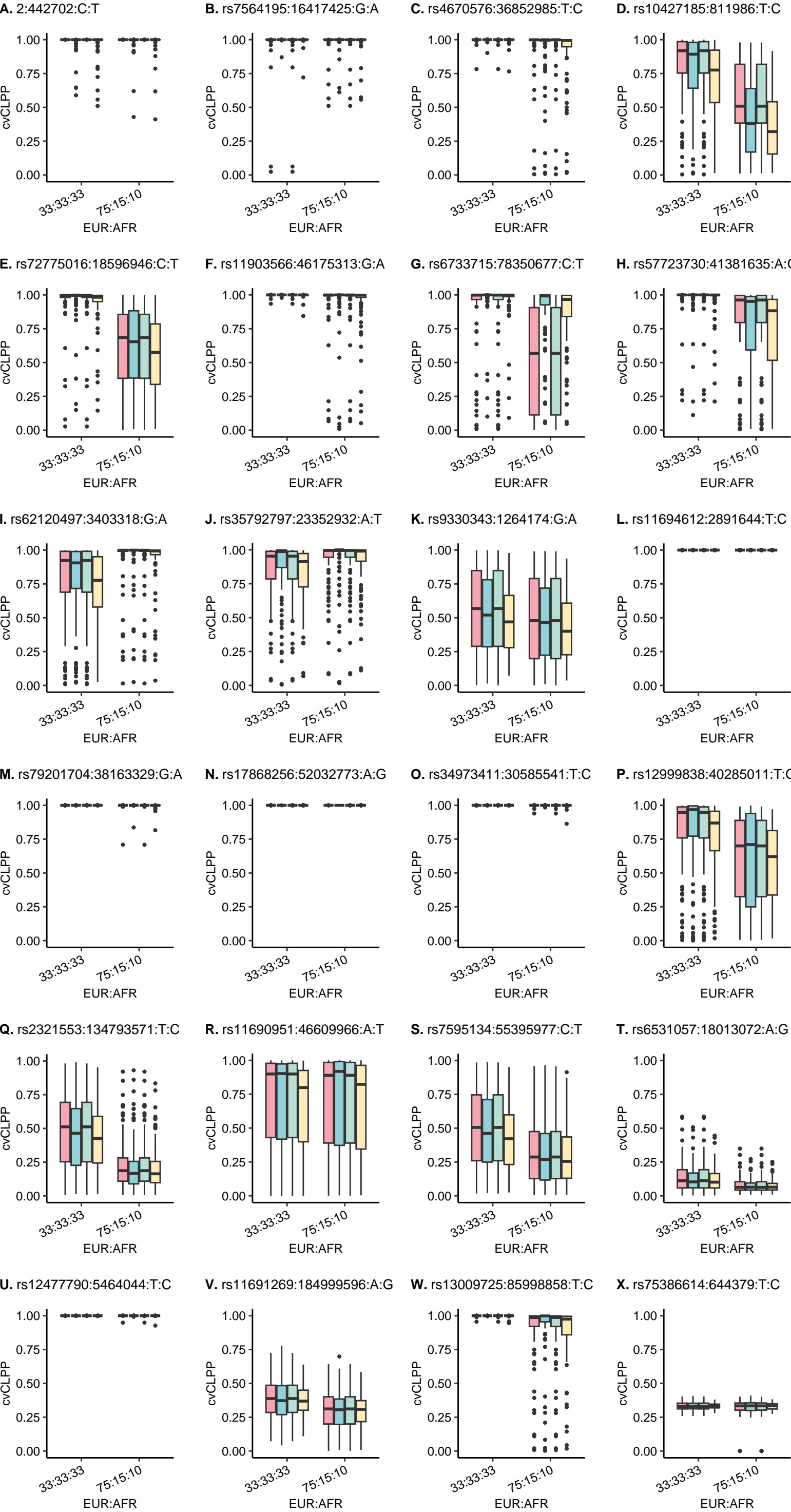

Supplement: S12 Fig — Two ancestry proportions were considered: 33/33/33 and 75/15/10 EUR:EAS:AFR, for a total N = 100000 in each setting. Conditional variant level CLPP is the probability the variant is colocalized, given that the locus is colocalized. (PDF) [file pgen.1012221.s012.pdf]

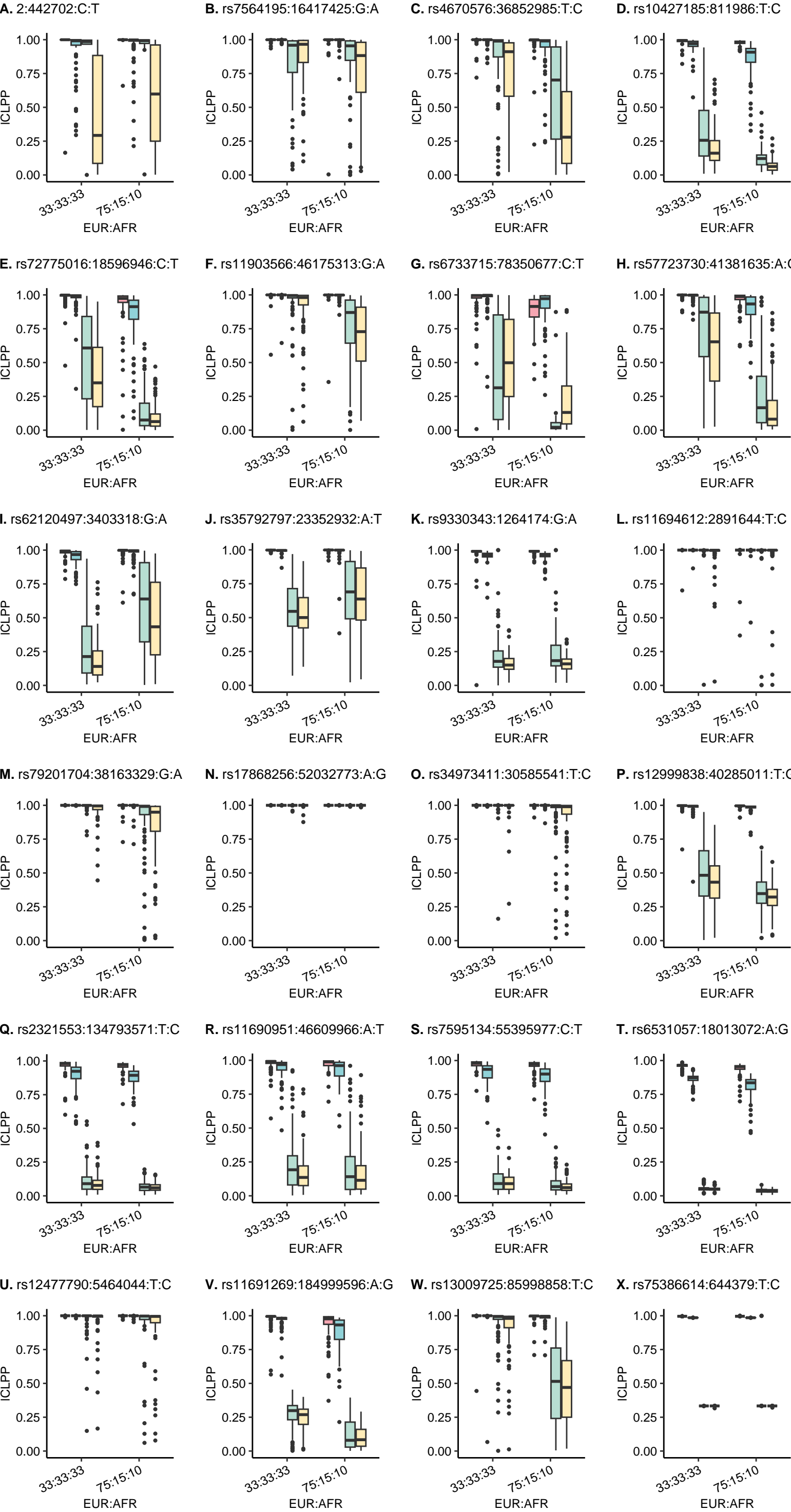

Supplement: S13 Fig — Two ancestry proportions were considered: 33/33/33 and 75/15/10 EUR:EAS:AFR, for a total N = 100000 in each setting. Locus level CLPP is the probability that the locus is colocalized. (PDF) [file pgen.1012221.s013.pdf]

A.

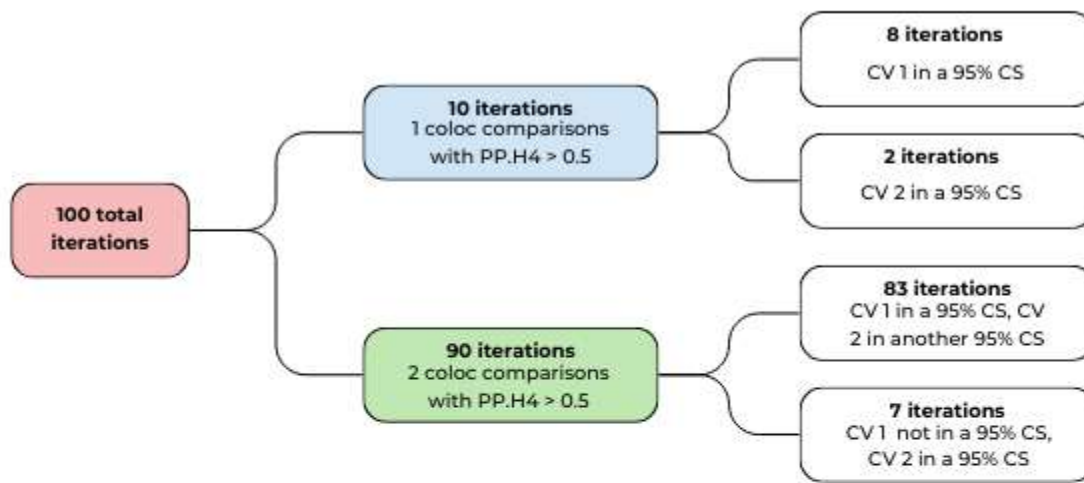

B.

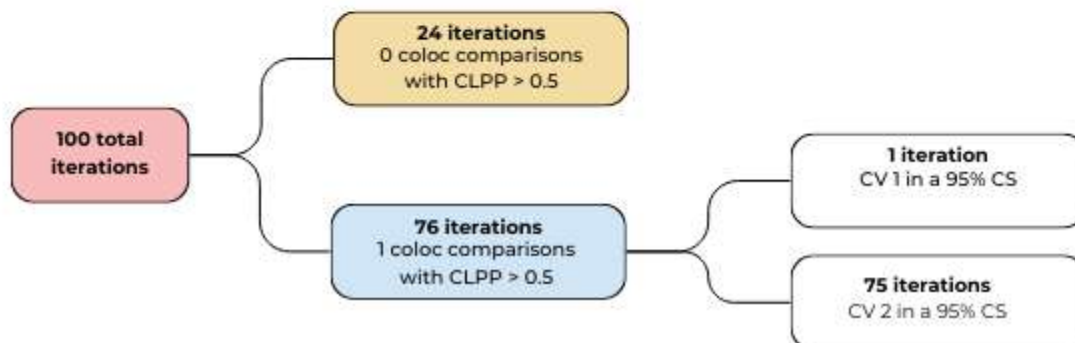

Supplement: S14 Fig — Multi-ancestry colocalization approaches were evaluated on chr2:191476292–191576292 (GRCh37), centered around causal variants rs10200534:191537142:C:A and, rs1023568:191515442:A:T by A. coloc_SuSiEx, B. eCAVIAR_SuSiEx. For coloc comparisons with a locus level CLPP > 0.5, a 95% colocalization credible set was constructed by ranking variants by conditional variant level CLPPs and summing until the cumulative CLPP exceeds 0.95. (PDF) [file pgen.1012221.s014.pdf]

A.

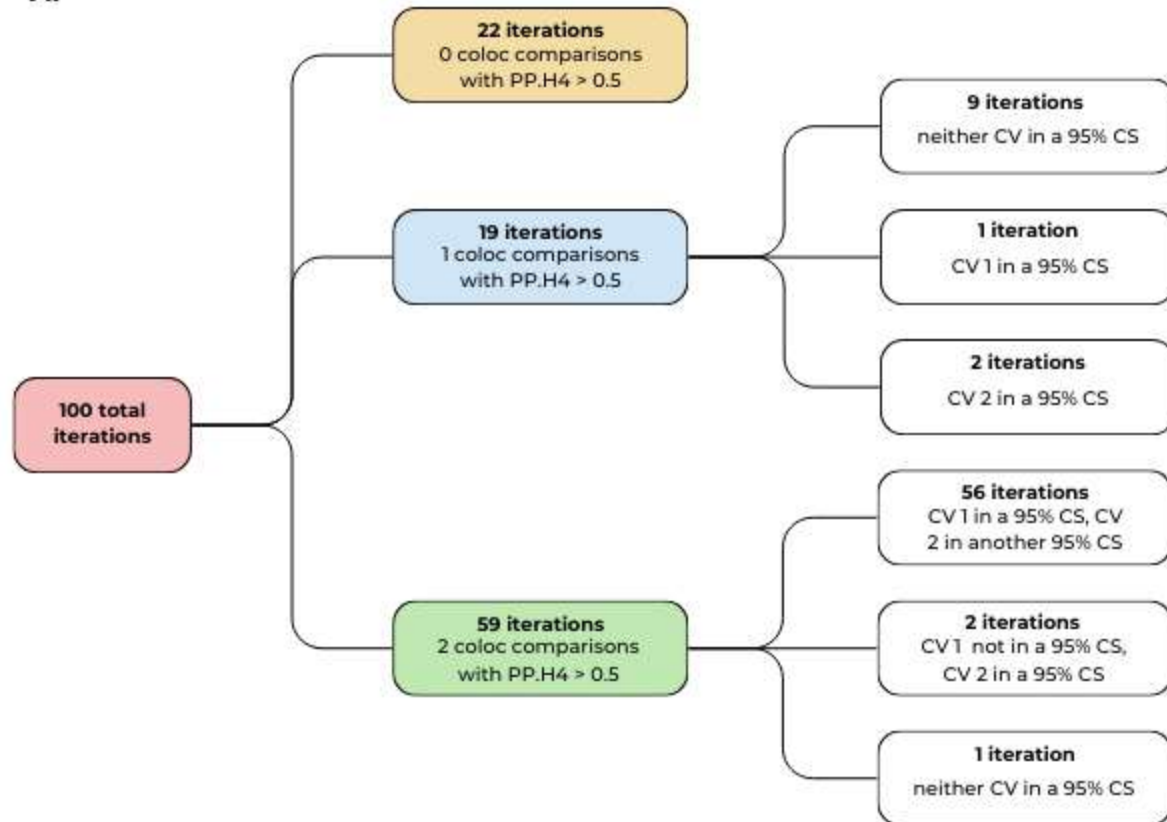

B.

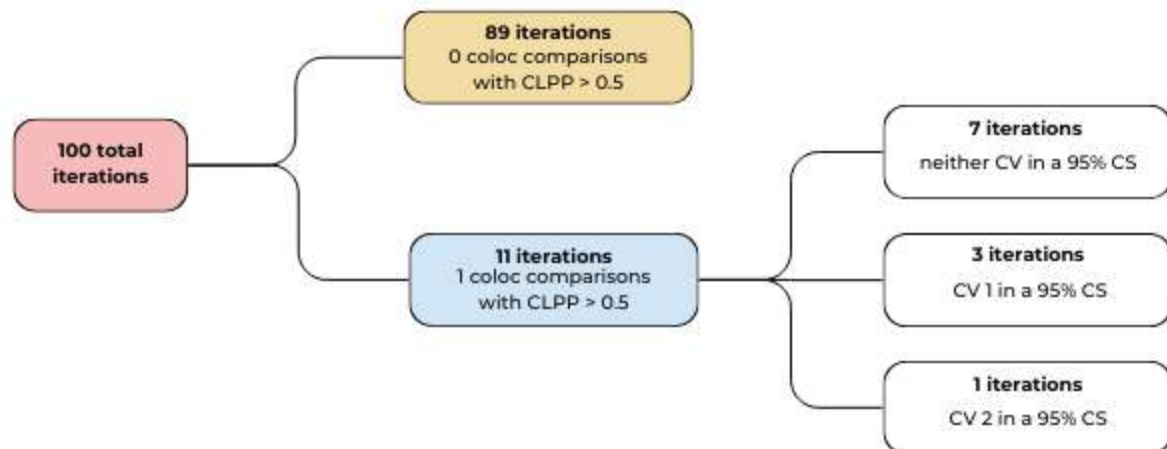

Supplement: S15 Fig — Multi-ancestry colocalization approaches were evaluated on chr2:171130765–171230765 (GRCh37), centered around causal variants rs59441748:171185685:C:A and rs716427:171175844:A:G by A. coloc_SuSiEx, B. eCAVIAR_SuSiEx. For coloc comparisons with a locus level CLPP > 0.5, a 95% colocalization credible set was constructed by ranking variants by conditional variant level CLPPs and summing until the cumulative CLPP exceeds 0.95. (PDF) [file pgen.1012221.s015.pdf]

A.

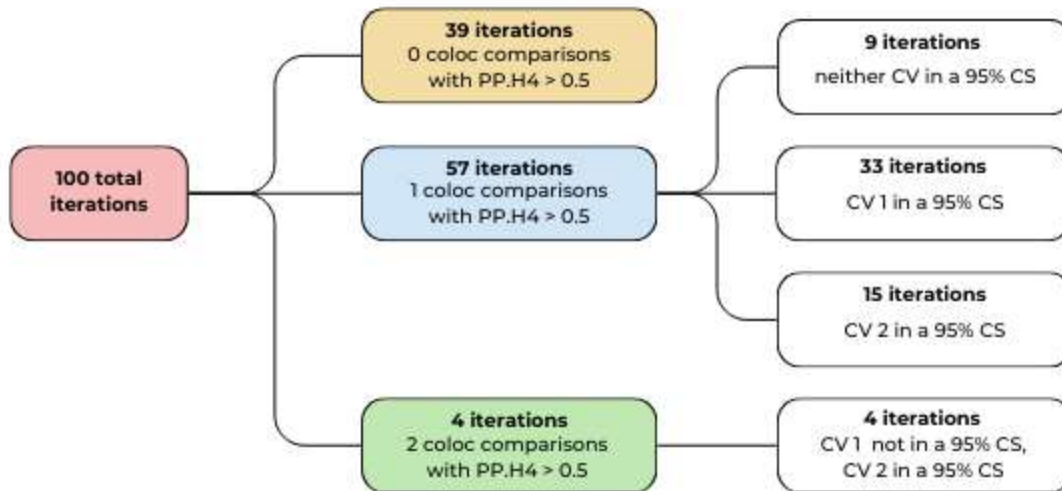

B.

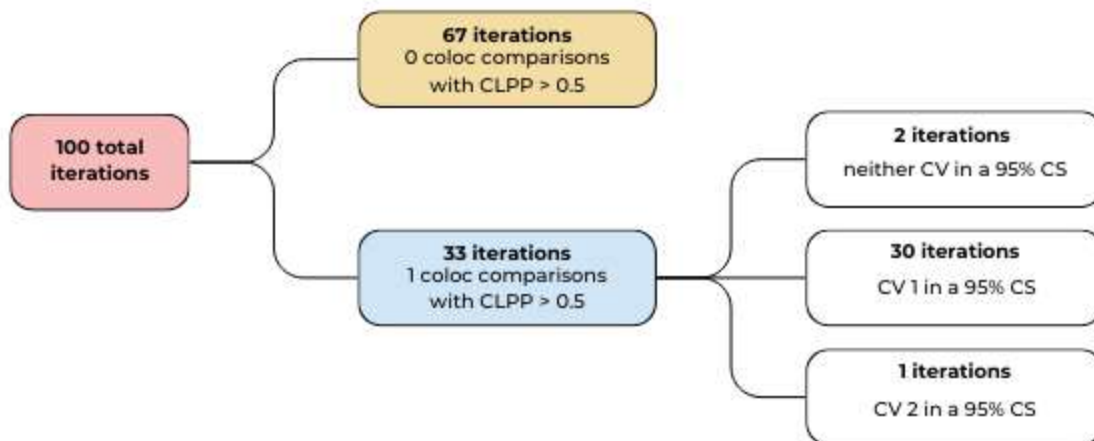

Supplement: S16 Fig — Multi-ancestry colocalization approaches were evaluated on 50/50 EUR:AFR datasets on chr2:17265320–17365320 (GRCh37), centered around causal variants rs71447280:17325432:A:G and rs60143916:17305207:G:A by A. coloc_SuSiEx, B. eCAVIAR_SuSiEx. For coloc comparisons with a locus level CLPP > 0.5, a 95% colocalization credible set was constructed by ranking variants by conditional variant level CLPPs and summing until the cumulative CLPP exceeds 0.95. (PDF) [file pgen.1012221.s016.pdf]

A.

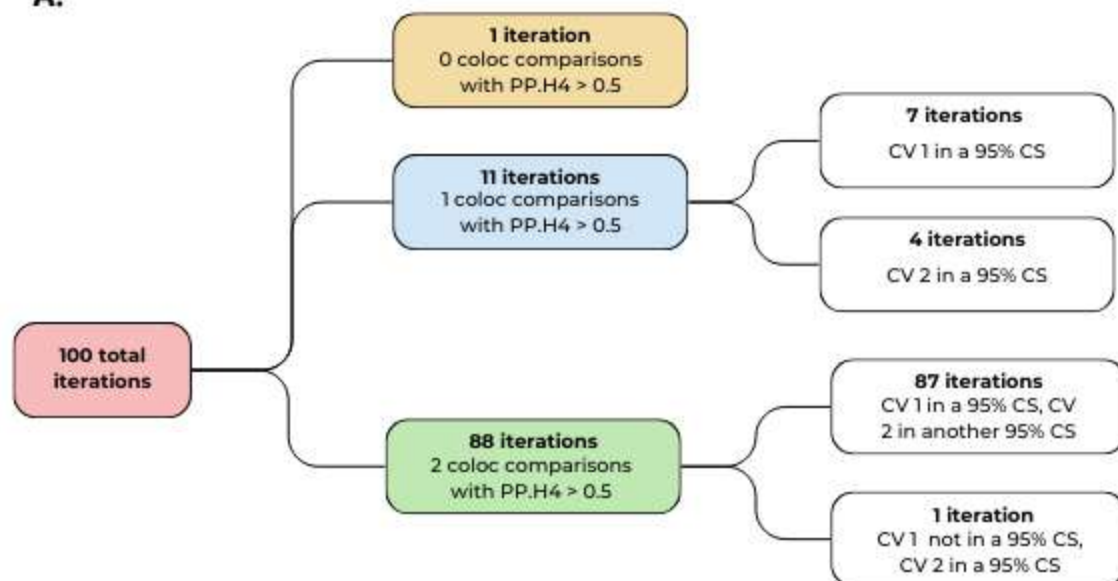

B.

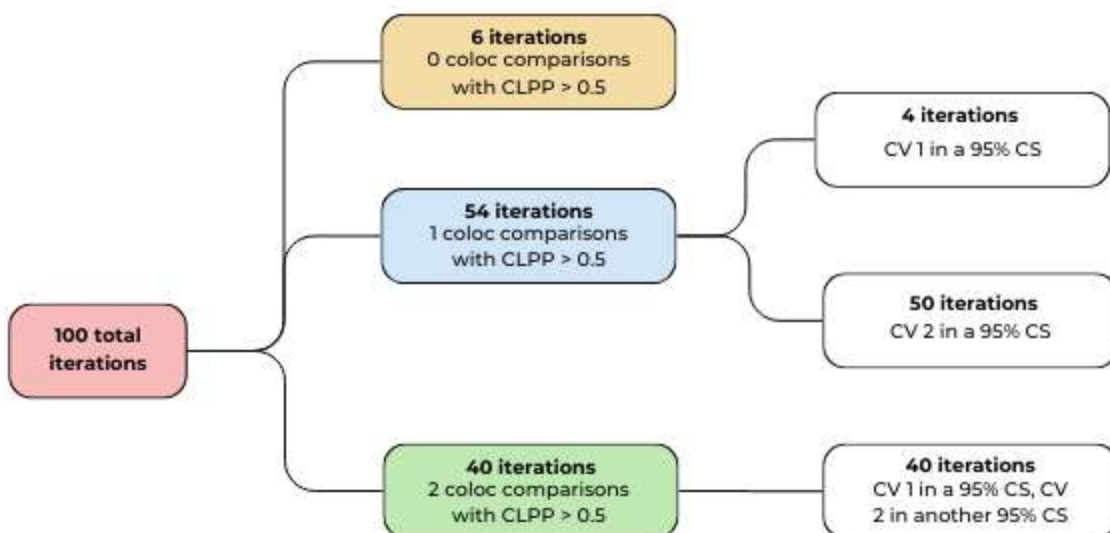

Supplement: S17 Fig — Multi-ancestry colocalization approaches were evaluated on 50/50 EUR:AFR datasets on chr2:67906038–68006038 (GRCh37), centered around causal variants rs142101372:67967864:A:C and rs1864572:67944212:T:C by A. coloc_SuSiEx, B. eCAVIAR_SuSiEx. For coloc comparisons with a locus level CLPP > 0.5, a 95% colocalization credible set was constructed by ranking variants by conditional variant level CLPPs and summing until the cumulative CLPP exceeds 0.95. (PDF) [file pgen.1012221.s017.pdf]

**B. rs7575324:1208845:A:G and rs35784786:1239516:C:T**

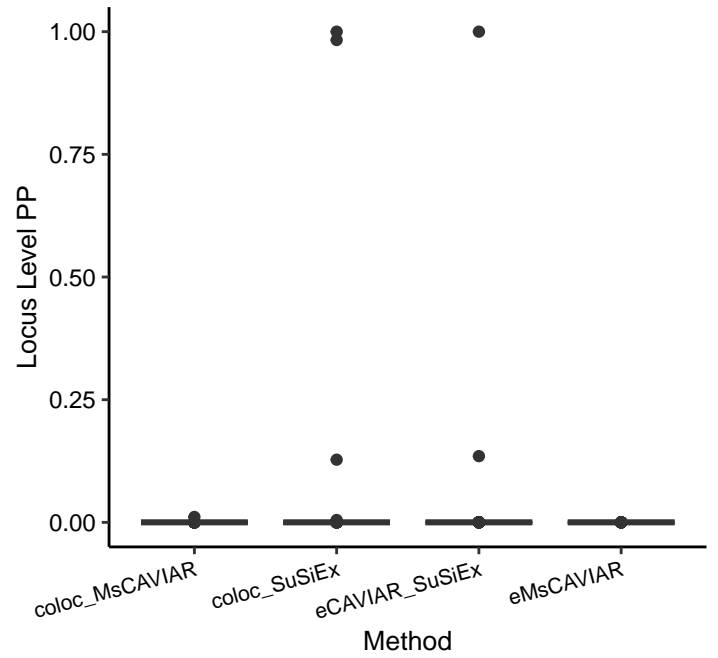

**D. rs823550:2465607:G:T and rs823551:2467773:A:G**

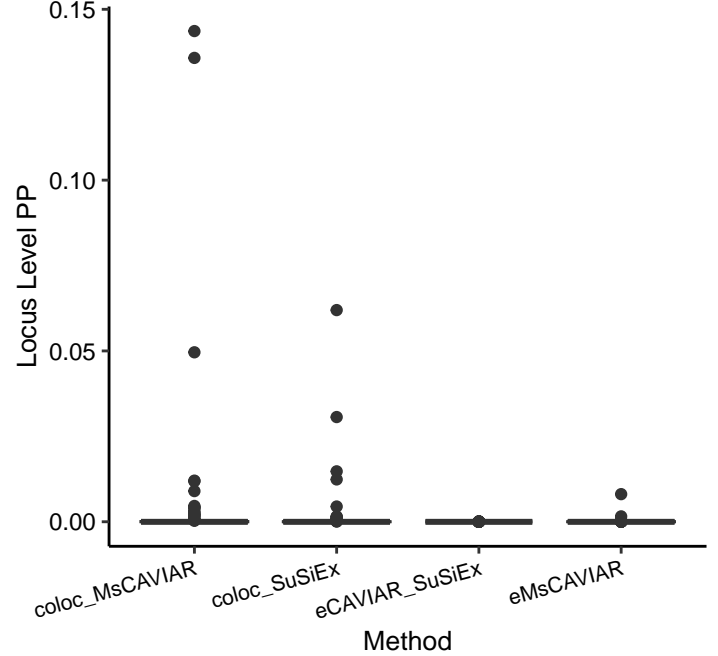

Supplement: S18 Fig — Multi-ancestry colocalization analysis approaches were applied to analyze EUR and AFR datasets on loci with distinct causal variants across traits, that is, on loci that are not colocalized across the traits under analysis. The locus level CLPP is the probability of colocalization within the locus. (PDF) [file pgen.1012221.s018.pdf]

**A.** 2:1002741:A:G and rs77602058:1046894:C:T

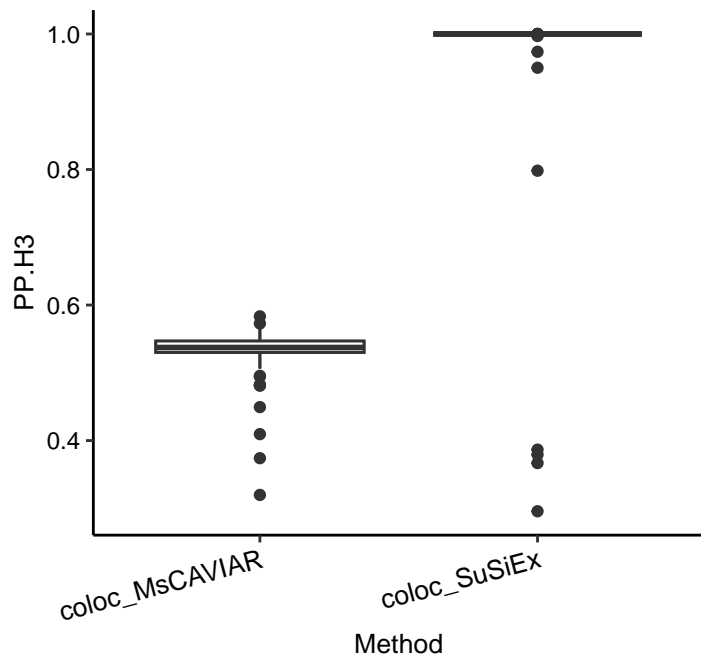

**B.** rs7575324:1208845:A:G and rs35784786:1239516:C:T

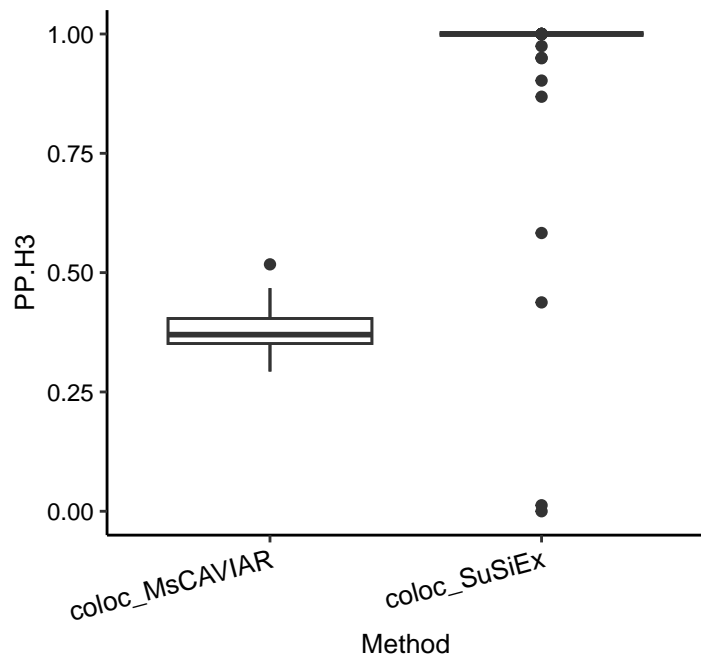

**C.** rs1967512:1423500:T:C and rs10519477:1431590:C:T

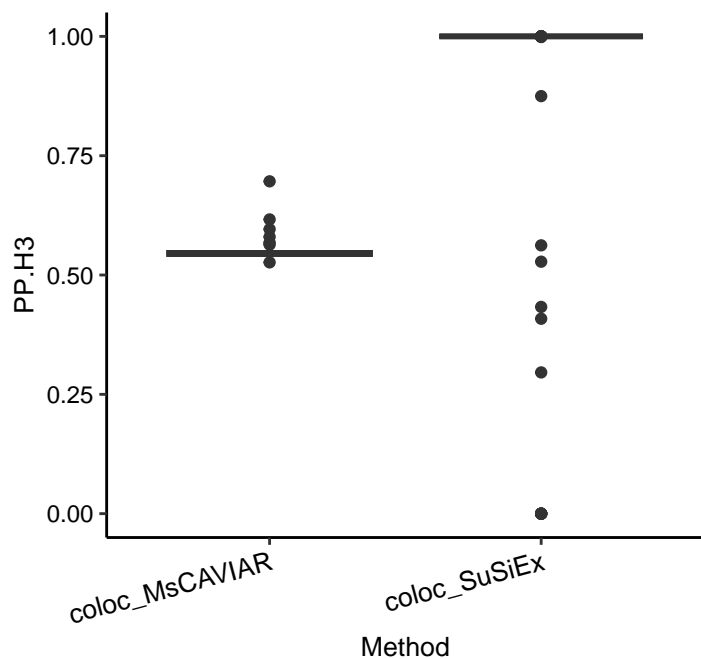

**D.** rs823550:2465607:G:T and rs823551:2467773:A:G

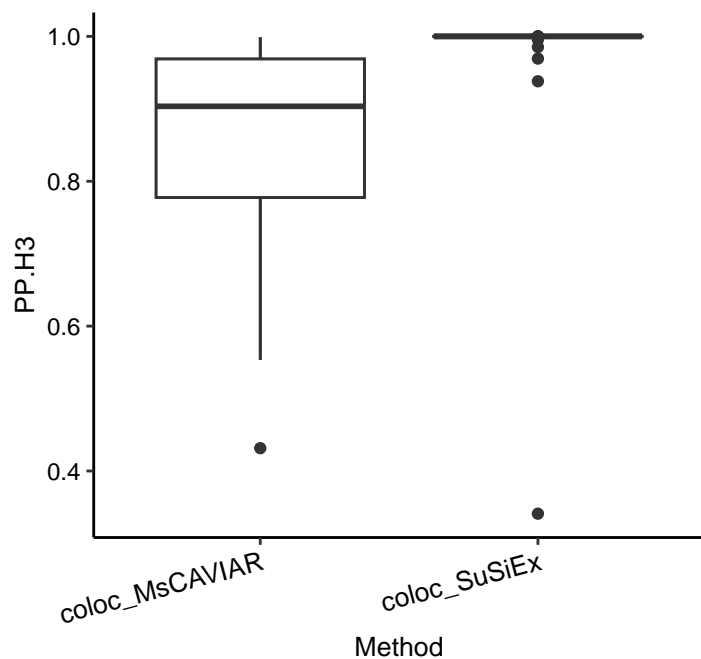

Supplement: S19 Fig — Multi-ancestry colocalization analysis approaches were applied to analyze EUR and AFR datasets on loci with distinct causal variants across traits, that is, on loci that are not colocalized across the traits under analysis. PP.H3 is the posterior probability corresponding to coloc’s third hypothesis, where two traits have an association signal at the same locus, but the association signals are explained by different causal variants. (PDF) [file pgen.1012221.s019.pdf]
