# Supplementary material for: Multi-ancestry colocalization approaches
Source: PLoS Genet. 2026 Jul 21;22(7):e1012221. doi: 10.1371/journal.pgen.1012221 (PMC13387578; doi:10.1371/journal.pgen.1012221)
Supplement: S5 Fig — Two ancestry proportions were considered: 50/50 and 80/20 EUR:AFR, for a total N = 100000 in each setting. Locus level CLPP is the probability that the locus is colocalized. (PDF) [file pgen.1012221.s005.pdf]

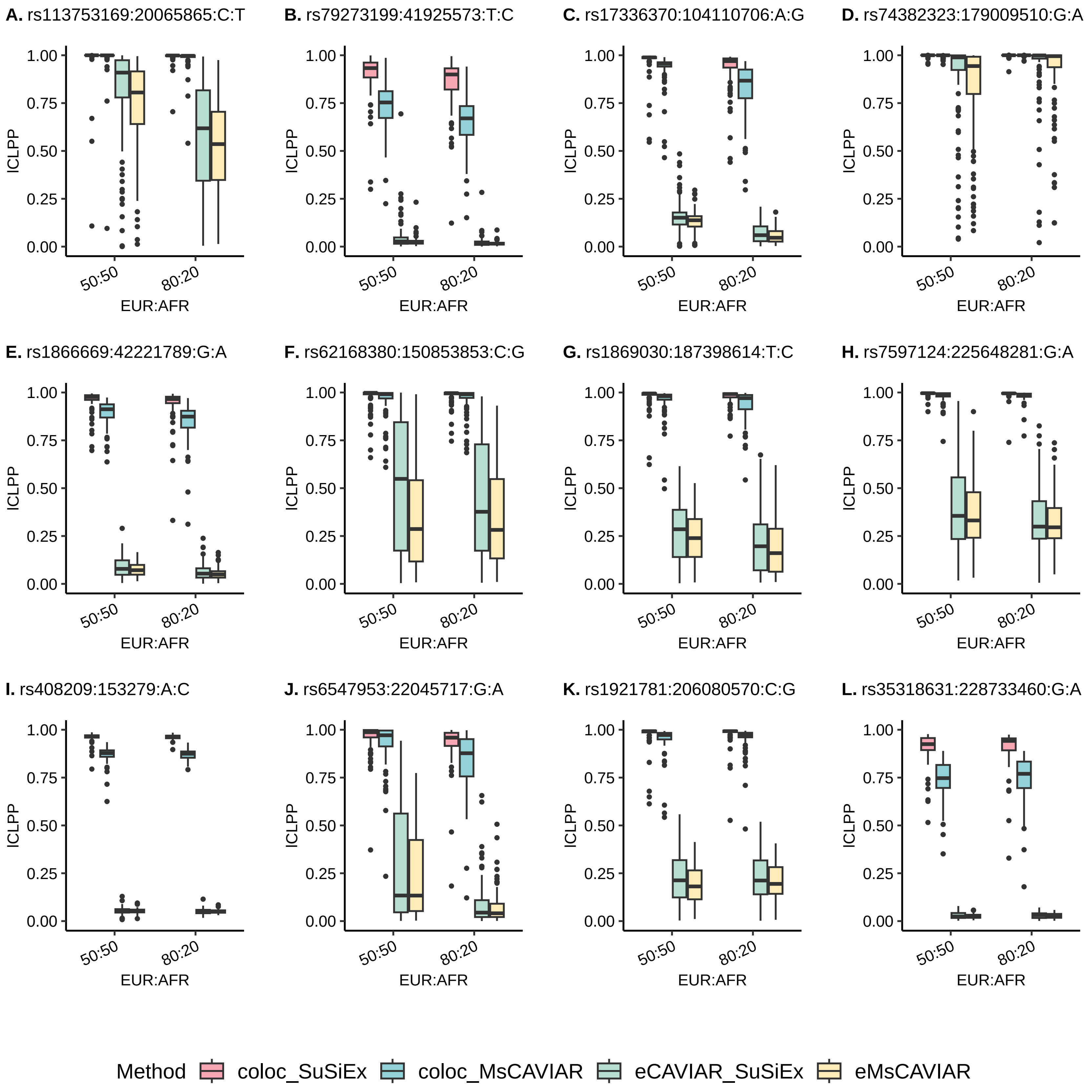

A. rs113753169:20065865:C:T
B. rs79273199:41925573:T:C
C. rs17336370:104110706:A:G
D. rs74382323:179009510:G:A
E. rs1866669:42221789:G:A
F. rs62168380:150853853:C:G
G. rs1869030:187398614:T:C
H. rs7597124:225648281:G:A
I. rs408209:153279:A:C
J. rs6547953:22045717:G:A
K. rs1921781:206080570:C:G
L. rs35318631:228733460:G:A
ICLPP
1.00
0.75
0.50
0.25
0.00
50:50
80:20
EUR:AFR
Method
coloc_SuSiEx
coloc_MsCAVIAR
eCAVIAR_SuSiEx
eMsCAVIAR
