# Supplementary material for: Multi-ancestry colocalization approaches
Source: PLoS Genet. 2026 Jul 21;22(7):e1012221. doi: 10.1371/journal.pgen.1012221 (PMC13387578; doi:10.1371/journal.pgen.1012221)
Supplement: S7 Fig — Two ancestry proportions were considered: 50/50 and 80/20 EUR:AFR, for a total N = 100000 in each setting. Coverage is the proportion of iterations the causal variant was included in the 95% credible set out of 100 iterations. (PDF) [file pgen.1012221.s007.pdf]

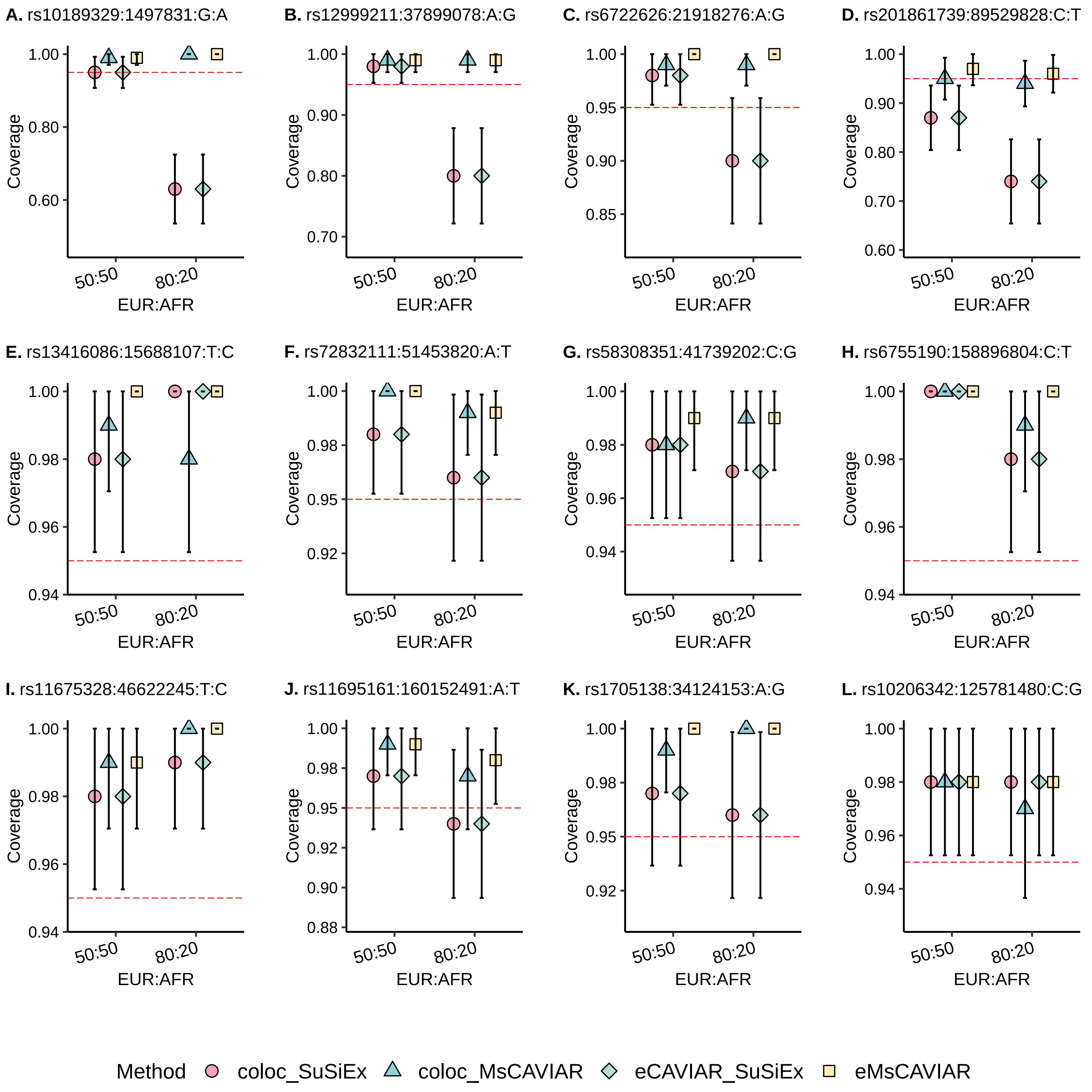

A. rs10189329:1497831:G:A
B. rs12999211:37899078:A:G
C. rs6722626:21918276:A:G
D. rs201861739:89529828:C:T
E. rs13416086:15688107:T:C
F. rs72832111:51453820:A:T
G. rs58308351:41739202:C:G
H. rs6755190:158896804:C:T
I. rs11675328:46622245:T:C
J. rs11695161:160152491:A:T
K. rs1705138:34124153:A:G
L. rs10206342:125781480:C:G
Coverage
50:50
80:20
EUR:AFR
Method
coloc_SuSiEx
coloc_MsCAVIAR
eCAVIAR_SuSiEx
eMsCAVIAR
